# Novel population of small tumour-initiating stem cells in the ovaries of women with borderline ovarian cancer

**DOI:** 10.1038/srep34730

**Published:** 2016-10-05

**Authors:** Irma Virant-Klun, Martin Stimpfel

**Affiliations:** 1 Department of Obstetrics and Gynaecology, University Medical Centre Ljubljana, 1000 Ljubljana, Slovenia

## Abstract

Small stem cells with diameters of up to 5 μm previously isolated from adult human ovaries indicated pluripotency and germinal lineage, especially primordial germ cells, and developed into primitive oocyte-like cells *in vitro*. Here, we show that a comparable population of small stem cells can be found in the ovarian tissue of women with borderline ovarian cancer, which, in contrast to small stem cells in “healthy” ovaries, formed spontaneous tumour-like structures and expressed some markers related to pluripotency and germinal lineage. The gene expression profile of these small putative cancer stem cells differed from similar cells sorted from “healthy” ovaries by 132 upregulated and 97 downregulated genes, including some important forkhead box and homeobox genes related to transcription regulation, developmental processes, embryogenesis, and ovarian cancer. These putative cancer stem cells are suggested to be a novel population of ovarian tumour-initiating cells in humans.

“Ordinary” ovarian cancer occurs when cells, comprising primarily epithelial cells in the ovarian surface epithelium, epithelial inclusion glands and even fimbria of the fallopian tube, begin to undergo uncontrolled growth and spread to other tissues and organs, leading to metastasis^1^,^2^,^3^,^4^,^5^,^6^,^7^. Ovarian cancer is highly resistant to therapy, which usually results in the remission of disease and is highly lethal^8^,^9^. It is difficult to diagnose ovarian cancer because of the lack of symptoms; therefore, it is generally diagnosed at an advanced stage (Grade 3 or 4) and usually corresponds to a poor prognosis for the patient^10^. Although borderline ovarian cancer arises from the same progenitor cells in the ovarian surface epithelium as “ordinary” ovarian cancer, its growth is much more controlled at the molecular level, and it is less aggressive than “ordinary” serous or mucinous ovarian cancers because of its still poorly understood molecular mechanisms. Therefore, borderline ovarian cancer is classified as an independent group of ovarian epithelial tumours^11^. Usually, it is diagnosed at an early stage (Grade 1) and can be successfully cured, most often by surgery^12^,^13^. Despite this, in some rare patients, borderline ovarian cancer can spread beyond the ovary to other tissues and organs^14^,^15^,^16^. In these patients, the disease behaves more like “ordinary” ovarian cancer, and a malignant tumour with a strong tendency to invade other tissues can form. In such cases, the disease is highly resistant to therapy and may be recurrent and lethal^17^,^18^,^19^.

Little data exists regarding the mechanisms that induce the manifestation of borderline ovarian cancer, especially the subsequent formation of invasive growths in other tissues and organs and malignant and resistant tumours in patients. Recent data showed that mutations of certain genes, such as *BRAF*, *KRAS*, *NRAS*, *USP9X,* and *EIF1AX*, which are involved in mTOR regulation, and various miRNAs may be related to the progression of borderline ovarian tumours to low-grade serous ovarian carcinoma^20^,^21^,^22^. However, the mechanisms are still poorly understood. The high resistance, remission and lethality of “ordinary” ovarian cancer is more commonly attributed to cancer stem cells^23^,^24^,^25^,^26^. It is becoming clear that different types of stem cells are involved in the manifestation of ovarian cancer, and such stem cells may cooperate with other types of cells. The data from different studies have also indicated the presence of stem cells in ovarian surface epithelium and their potential involvement in the manifestation of ovarian cancer^27^,^28^. Notwithstanding such previous efforts, there is no generally accepted population of ovarian cancer-initiating stem cells in human ovaries. Different studies have reported ovarian tumour-initiating stem cells of different phenotypes based on the expression of some established markers, such as CD133 and ALDH1, which may complicate clinical applications^29^,^30^,^31^,^32^,^33^,^34^. In addition, most data correspond to ovarian cell lines, which do not necessarily reflect the situation *in vivo*.

To our knowledge, until now, there has been no reported experimental data identifying a direct relationship between borderline ovarian cancer and stem cells in humans. Here, we report a novel population of small putative cancer stem cells from borderline ovarian cancer, which, opposite to such cells in “healthy” ovaries, spontaneously form tumour-like structures *in vitro*, express several genes related to pluripotency and germinal lineage, and significantly differ from a similar population of small stem cells from “healthy” ovaries in terms of their gene expression profile, including forkhead box and homeobox genes, which are related to transcription regulation, developmental processes, embryogenesis, and cancer. These small putative cancer stem cells may be a novel population of tumour-initiating cells in borderline ovarian cancer in humans and should be further studied.

## Results

Initially, we observed a random finding. During part of our ovarian stem cell research, we received an ovarian cortex biopsy from a patient who had been surgically treated for a “benign” tumour. We carefully observed the ovarian tissue under an inverted microscope and found that it was different from all tissue samples that had been previously retrieved in our research of stem cells from “healthy” ovaries. Just after surgical retrieval, we found several small round cells and tumour-like structures in the tissue. After the ovarian tissue was enzymatically digested and the cells were cultured, we found specific small round cells, which were very similar to small putative stem cells identified in “healthy” adult human ovaries in our previous research^35^,^36^,^37^,^38^,^39^,^40^. However, in this woman, these small cells were different than all previous samples from other women: they highly proliferated and spontaneously formed tumour-like structures in the ovarian tissue just after surgical retrieval and in a cell culture.

This phenomenon captured our attention because the histopathological diagnosis specifically revealed borderline ovarian cancer in this patient. Therefore, we continued the investigation by studying small tumour-forming cells from the ovaries of eight patients with borderline ovarian cancer for comparison with a similar population of small cells from the “healthy” ovaries of three women without cancer.

### Specific small cells and tumour-like structures in the ovarian tissue of women with borderline ovarian cancer just after surgical retrieval

**Figure 1:**
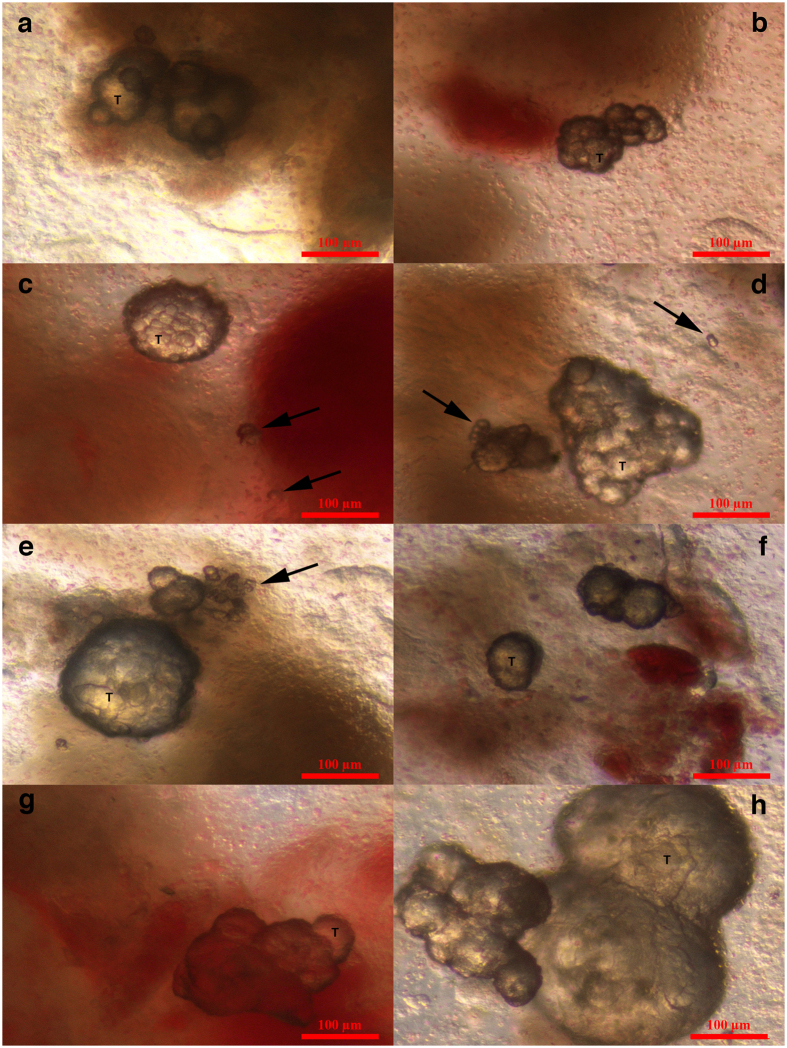
Small putative cancer stem cells with diameters of up to 5 μm forming tumour-like structures in the ovarian cortex tissue of one woman with borderline ovarian cancer just after surgical retrieval. (**a–h**) Small putative cancer stem cells (arrows) forming tumour-like structures (T) in borderline ovarian cancer. In the vicinity of the tumour-like structures are blood vessels and erythrocytes. (**h)** The tumour-like structures are spread by tiny blood vessels. Legend: (**a–h**) Inverted microscope, Hoffman illumination. Scale bar, 100 μm (**a**–**h**).

**Figure 2:**
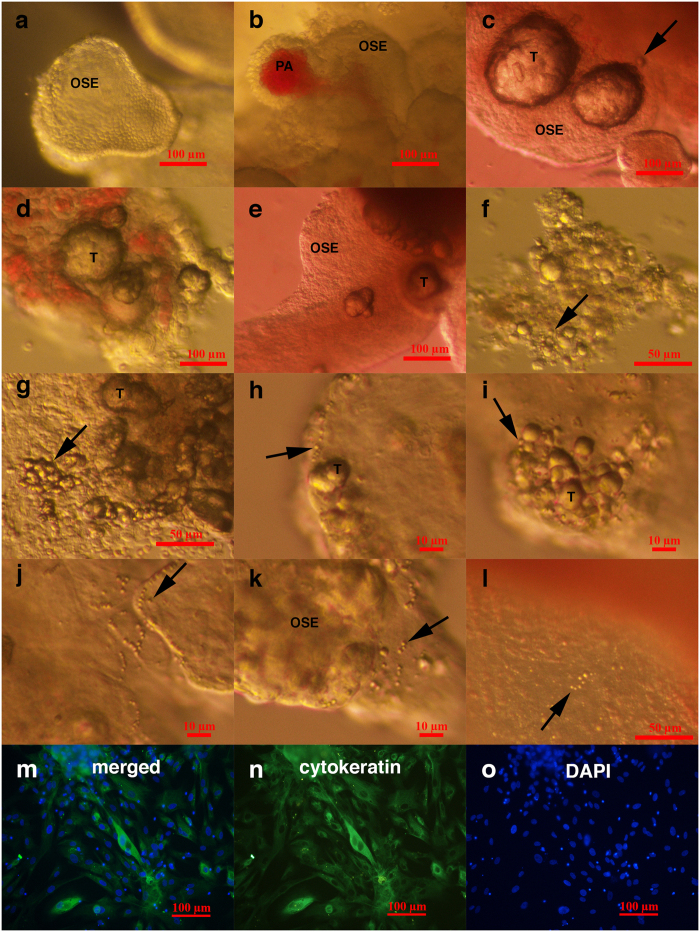
Typically shaped ovarian surface epithelium (OSE) and small putative cancer stem cells forming tumour-like structures in the ovarian cortex tissue of three women with borderline ovarian cancer in comparison to “healthy” ovarian tissue just after surgical retrieval. (**a–c**) Typically shaped ovarian surface epithelium (OSE). (**b**) Ovarian surface epithelium forming papillae (PA) that are vascularised. (**c–k**) Small putative cancer stem cells (arrows) forming tumour-like structures (T). (**l**) Small stem cells (arrow) in “healthy” ovarian cortex tissue that do not form tumour-like structures. (**m–o**) Ovarian cells positively stained for cytokeratin (green), as revealed by immunocytochemistry. Legend: (**a-l**) Inverted microscope, Hoffman illumination; (**m–o**) fluorescence microscope. Scale bar, 10 μm (**h–k**), 50 μm (**f,g,l**), 100 μm (**a–e,m–o**).

In all eight patients, we observed small round cells with diameters of up to 5 μm in the ovarian cortex tissue just after surgical retrieval, as observed in Figs 1c–e and 2c,f-k. These cells looked like small golden “nuggets” with a slight yellow appearance that shined or reflected a special light. They were of different sizes within this range, and in some places, they formed tumour-like structures, as observed in the same figures (Figs 1a–h and 2c–e,g–i). The whole ovarian tissue was expanded by tumour-like structures of different sizes, and the tissue expressed the “cauliflower” structure (Fig. 2d). There were also several traces of small blood vessels appearing around the tumour-like structures (Fig. 1b,c,f–h). A proportion of tumour-like structures were mineralized and of solid consistency. Adjacent to the tissue, the ovarian surface epithelium exhibited a typical shape (see Fig. 2a–c,e), and small round cells were attached to the epithelial cells (Fig. 2f-k), as previously observed in ovarian surface epithelium retrieved by laparoscopic brushing of “healthy” ovaries^41^. This tumour-like formation has not been observed in the ovarian tissue of any “healthy” women (Fig. 2l). The cytoplasm of the epithelial cells was positively stained for cytokeratin (Fig. 2m–o), an epithelial marker that is known to be expressed in ovarian epithelial cancers^42^.

### Cell colonies and tumour-like structures *in vitro* after enzymatic degradation of ovarian tissue

**Figure 3:**
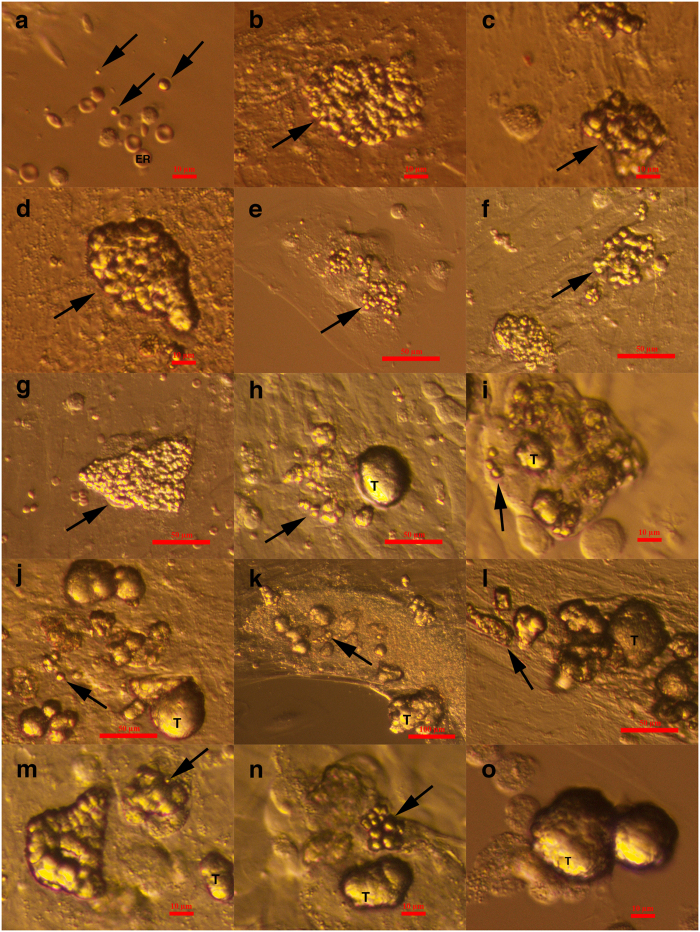
Small putative cancer stem cells from three women with borderline ovarian cancer forming tumour-like structures during *in vitro* culture in comparison to small stem cells from “healthy” ovarian tissue from one woman just after enzymatic digestion. (**a**) Small stem cells (arrows) among erythrocytes (ER) from “healthy” ovarian cortex tissue after enzymatic digestion that do not form any tumour-like structures. (**b–o**) Small putative cancer stem cells (arrows) from “cancerous” ovarian cortex tissue during *in vitro* culture after enzymatic digestion of tissue that aggregate into tumour-like structures (T). Inverted microscope, Hoffman illumination, scale bar, 10 μm (**a–d,i,m–o**), 50 μm (**e–h,j,l**), 100 μm (**k**).

**Figure 4:**
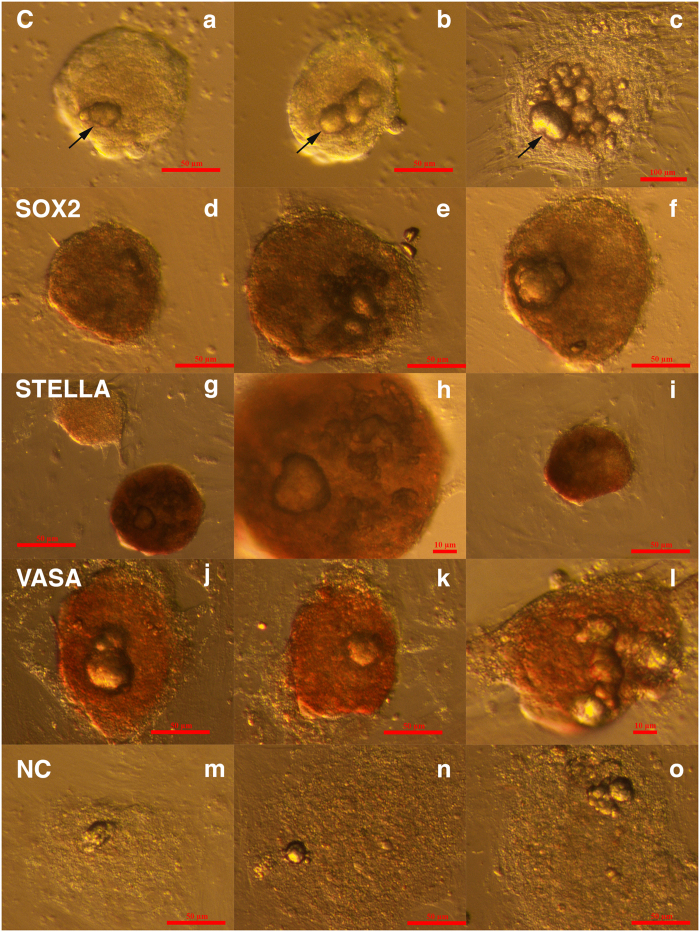
Cell colonies with tumour-like structures from two women with borderline ovarian cancer, which developed *in vitro* and stained positively for markers of pluripotency and germinal lineage, SOX2, STELLA/DPPA3, and VASA/DDX4, in comparison to the non-stained negative control according to immunocytochemistry by DAB. **(a–c)** Cell colonies, including tumour-like structures (arrows), that did not undergo immunocytochemistry (scale bar: 1^st^ and 2^nd^ photo 50 μm, 3^rd^ photo 100 μm); **(d–f)** Cell colonies and incorporated tumour-like structures that were positively stained for SOX2 marker of pluripotency (brown) (scale bar: 50 μm); **(g–i)** Cell colonies with tumour-like structures that were positively stained for pluripotency and primordial germ cell-related marker STELLA (brown); **(g)** One positively stained and one non-stained cell colony (scale bar: 1^st^ and 3^rd^ photo 50 μm, 2^nd^ is magnified 1^st^ 10 μm); **(j–l)** Cell colonies with tumour-like structures that were positively stained for germinal lineage-related marker VASA (brown) (scale bar: 1^st^ and 2^nd^ photo 50 μm, 3^rd^ photo 10 μm). **(m–o)** Cell colonies with tumour-like structures that served as a negative control (NC) and did not stain for the above markers (scale bar: 50 μm).

One cell culture was carried out for each woman. After two-step enzymatic degradation of the ovarian tissue using collagenase and hyaluronidase and filtering through a 70-μm cell strainer, the ovarian cells were cultured in supplemented DMEM/F-12 culture medium on gelatine as an adherent layer, as described in our previous work on “healthy” non-cancerous ovaries^40^. Initially, small round cells appeared among the blood and epithelial cells (Fig. 3a). In the following days, the cells attached to the gelatine, and cell colonies began to appear. The cell colonies that formed from the tissue of women with borderline ovarian cancer were quite comparable to those formed in the ovarian cell cultures from “healthy” women. Like in “healthy” ovaries, we observed typical small, round cells with diameters of up to 5 μm in the cell cultures of all eight women with borderline ovarian cancer (Fig. 3b–n). While unlikely in cell cultures from “healthy” ovaries, in cell cultures from borderline ovarian cancer, these small round cells proliferated (Fig. 3b–g) and spontaneously formed tumour-like structures (Fig. 3h–o). The tumour-like structures formed separately (Fig. 3h–o) or inside the cell colonies (Fig. 4a–o). The tumour-like structures were characterized by abnormal growth and aggregation of small round cells resembling small human tumours according to their phenotype in clinical practice. It is interesting that the tumour-like structures that formed *in vitro* started to mineralize spontaneously after a period of time. However, in cell cultures from “healthy” ovaries, there were no tumour-like structures despite the presence of highly similar populations of small round cells.

### Time-lapse microscopy of tumour-like structures from cell culture after enzymatic degradation of ovarian tissue

In one patient with borderline ovarian cancer, the ovarian cell culture, which included small round cells and tumour-like structures, was exposed to time-lapse monitoring. As shown in the attached videos (Supplemental Videos 1 and 2), it is possible to observe that the tumour-like structures grew by aggregation of small round cells. It was not possible to follow single round cells using the time-lapse camera because these cells were too small, and we observed them as quite dormant when removed from other cells in a culture, representing the ovarian “niche”.

### Expression of markers related to pluripotency and germinal lineage in cell cultures after enzymatic degradation of ovarian tissue

We focused on markers related to pluripotency and germinal lineage. Figure 4a–c shows the ovarian cell culture from borderline ovarian cancer, including the tumour-like structures that did not undergo immunocytochemistry. In the ovarian cell cultures stained by the DAB procedure, a proportion of cell colonies, including tumour-like structures and single cells with diameters of up to 5 μm, were positively brown stained for pluripotency-related marker SOX2 (Fig. 4d–f), primordial germ cell marker STELLA/DPPA3 (Fig. 4g–i) and germinal lineage-related marker VASA/DDX4 (Fig. 4j–l). In all these figures, it can be observed that both the tumour-like structures and the surrounding cells were positively stained for the markers that were assessed. There was, however, a proportion of cell colonies that did not stain positively for these markers (Fig. 4g). In addition, the negative controls did not stain for these markers (Fig. 4m–o).

**Figure 5:**
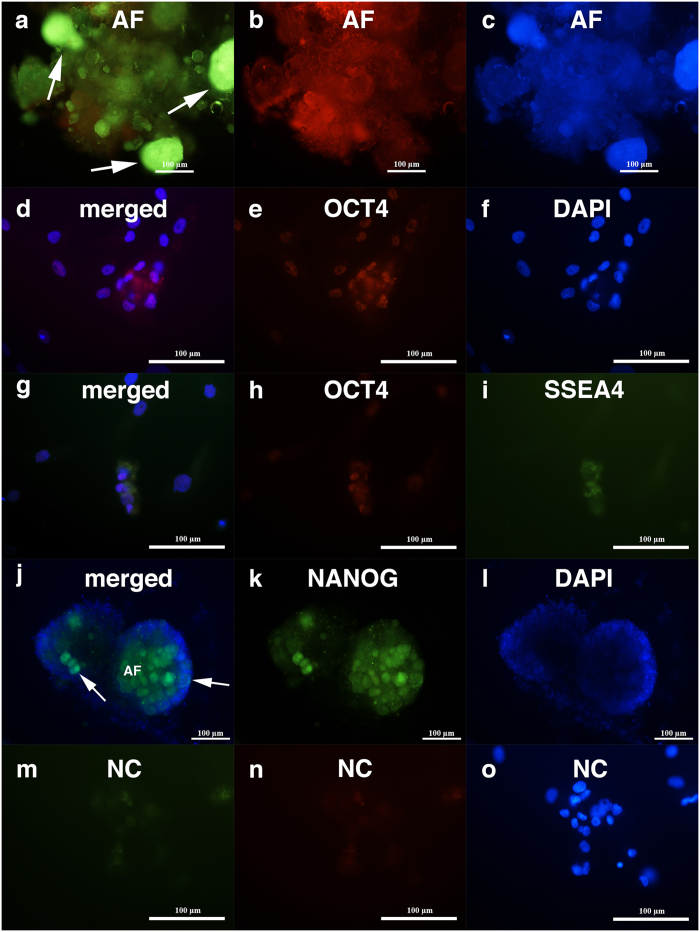
Small putative cancer stem cells and tumour-like structures from two women with borderline ovarian cancer, which developed *in vitro* and stained positively for markers related to pluripotency, OCT4, SSEA-4 and NANOG, in comparison to a non-stained negative control according to immunocytochemistry by immunofluorescence. **(a–c)** Autofluorescence (arrows) of mineralized tumour-like structures from borderline cancer (AF); **(d–f)** Nuclei of small putative cancer stem cells, which filled almost the entire cell volumes and were positively stained for OCT4 marker (red); **(g–i)** Double-staining of small putative cancer stem cells revealed positive nuclear staining for OCT4 (red) and surface staining for SSEA-4 (SSEA4). **(j–l)** Tumour-like structures were positively stained for NANOG (green). Among the mineralized tumour-like structures that were autofluorescent (AF) and did not express the outlined cells with DAPI-stained (blue) nuclei, there were also tumour-like structures that were composed of outlined cells with clearly observed nuclei (arrows). **(m–o)** Non-stained small putative cancer stem cells served as a negative control (NC). Fluorescence microscope, scale bar, 100 μm.

The ovarian tissue from the borderline ovarian cancer patients expressed strong autofluorescence, especially the mineralized tumour-like structures (Fig. 5a–c), when observed under a fluorescence microscope. This has also been observed in “ordinary” ovarian cancer by other groups and has been proposed as a potential diagnostic tool for cancer^43^. Nevertheless, we performed immunofluorescent staining of ovarian cell cultures, including the tumour-like structures, although the DAB staining was preferred. The immunofluorescent staining revealed positivity for pluripotency-related markers OCT4 (Fig. 5d–i), SSEA-4 (Fig. 5g–i) and NANOG (Fig. 5j–l) in a proportion of the cell colonies that had formed in the borderline ovarian cancer cell cultures. For the markers OCT4 and NANOG, nuclear positivity was observed, while marker SSEA-4 was expressed on the cell surfaces. Figure 5d–i shows the positivity of single cells. The nuclei of small putative cancer stem cells, which filled almost the entire cell volumes, were positively stained red for OCT4 (Fig. 5d–f); double-staining of small putative cancer stem cells revealed positive nuclear staining for OCT4 (red) and surface staining for SSEA-4 (green) (Fig. 5g–i). Figure 5j–l shows the positivity of tumour-like structures for NANOG (green). Some tumour-like structures were still composed of outlined cells with DAPI-stained nuclei and were positively stained for NANOG, while other tumour-like structures had already mineralized and were not composed of outlined cells with DAPI-stained nuclei and expressed autofluorescence, as observed in Fig. 5j–l. The negative control did not stain positively (Fig. 5m–o).

These data indicate that the small round cells that formed tumour-like structures were putative cancer stem cells.

### Transmission electron microscopy of tumour-like structures from cell culture after enzymatic degradation of ovarian tissue

**Figure 6:**
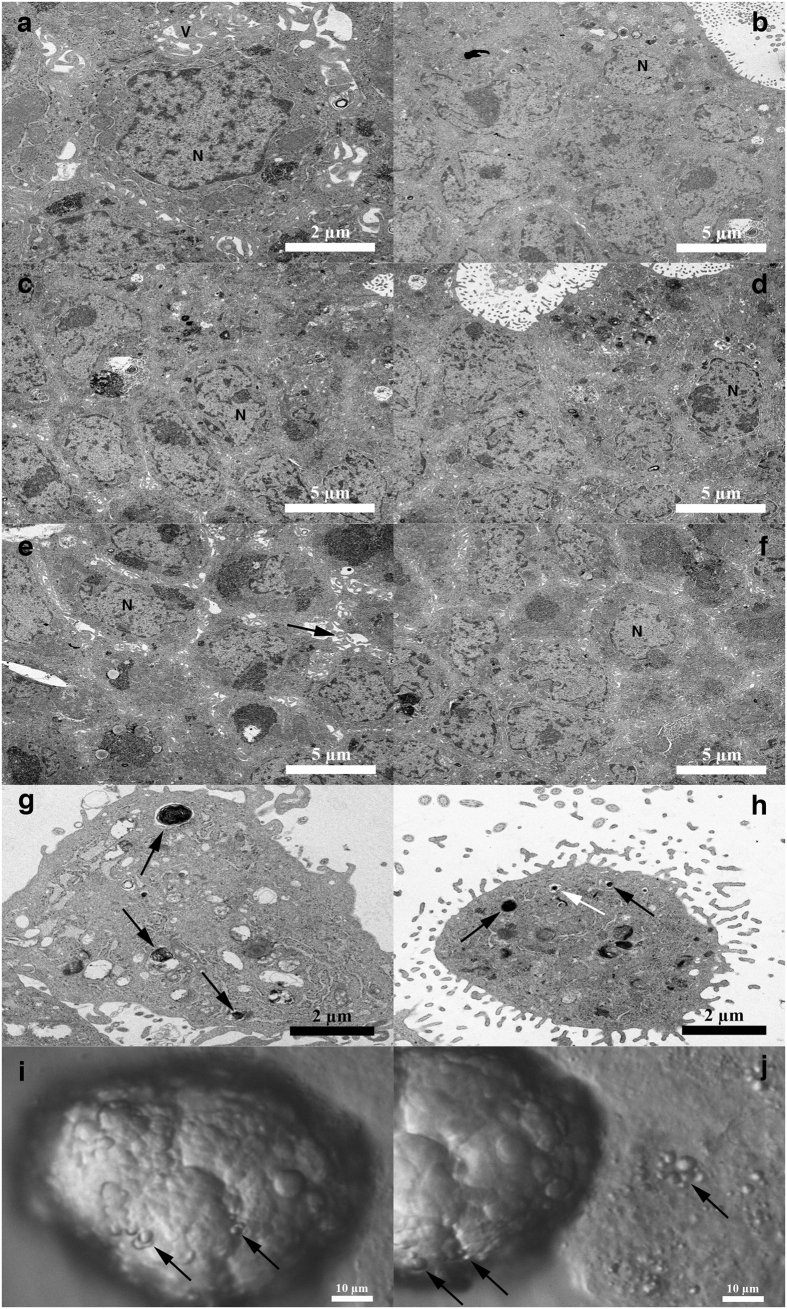
Ultrastructure of tumour-like structures from one woman with borderline ovarian cancer revealed by transmission electron microscopy. (**a**) Cells inside the tumour-like structure had diameters of approximately 5 μm with irregularly shaped nuclei that filled almost the entire cell volume and were covered by microvilli; (**b–d**) Solid tumour-like structure composed of tightly packed small cells with diameters of approximately 5 μm and with nuclei (N) filling almost the entire cell volumes. (**e,f**) Tumour-like structure composed of small cells with diameters of approximately 5 μm and with nuclei (N) filling almost the entire cell volumes and that are in the process of aggregation by their microvilli (arrow) and contents. (**g,h**) Small tumour-like structures with locations of mineralization (arrows) and covered by villi. (**i,j**) Growth of tumour-like structures from small putative cancer stem cells (arrows) were clearly observed also during cell culture *in vitro* (the same structure photographed at different times). Scale bar, 2 μm (**a,g,h**), 5 μm (**b–f**), 10 μm (**i,j**).

In one patient with borderline ovarian cancer, the tumour-like structures were manually taken from ovarian cell cultures, sectioned and then monitored by transmission electron microscopy (TEM). It can be clearly observed that tumour-like structures were formed from non-differentiated round cells with diameters of up to 5 μm and with nuclei filling almost the entire cell volumes (Fig. 6a,b). The nuclei of these cells were not round (Fig. 6a,b). In some sections, the small cells were tightly packed in a solid structure (Fig. 6c,d), while in other sections, the cells aggregated in a tumour-like structure by their microvilli (Fig. 6e,f). The tumour-like structures and the aggregating small cells were covered by several microvilli (Fig. 6e–h). The small cells retained their integrity in the tumour-like structures and might represent the small putative cancer stem cells forming tumour-like structures in cell cultures. In some tissue sections, mineralization was clearly observed and is visible in Fig. 6g,h. In Fig. 6i,j, the tumour-like structure and small round cells can be observed under an inverted microscope (magnification 400x); small round cells appear to attach to the tumour-like structure, as indicated by arrows.

### Sorting of small putative stem cells and tumour-like structures from ovarian cell cultures and microarray analysis

**Figure 7:**
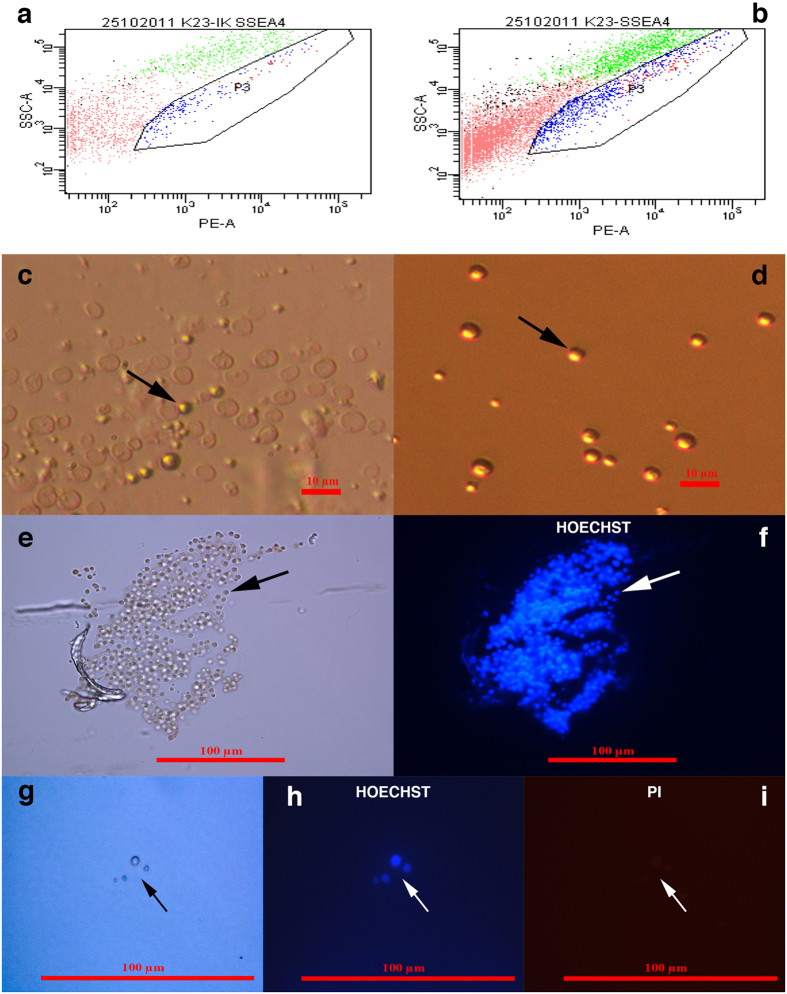
FACS sorting of small putative stem cells from healthy ovaries and their Hoechst 33258 and propidium iodide (PI) staining. **(a)** Dot plot of FACS-isotype control (IK); **(b)** Dot plot of FACS-sample; (**c**) Small putative stem cells (arrow) in enzymatically digested ovarian tissue; **(d,e)** Small putative stem cells (arrows) sorted from “healthy” ovarian tissue; **(f)** Blue nuclei filling almost the entire cell volumes of the small putative stem cells stained by Hoechst 33258; (**g**) Small putative stem cells observed under a light microscope (magnification 1000x) (arrow), (**h**) the nuclei of small putative stem cells stained blue by Hoechst 33258 (arrow), and (**i**) viable small putative stem cells that did not stain red by propidium iodide (PI) staining. Scale bar, 10 μm (**c,d**), 100 μm (**e–i**).

**Figure 8:**
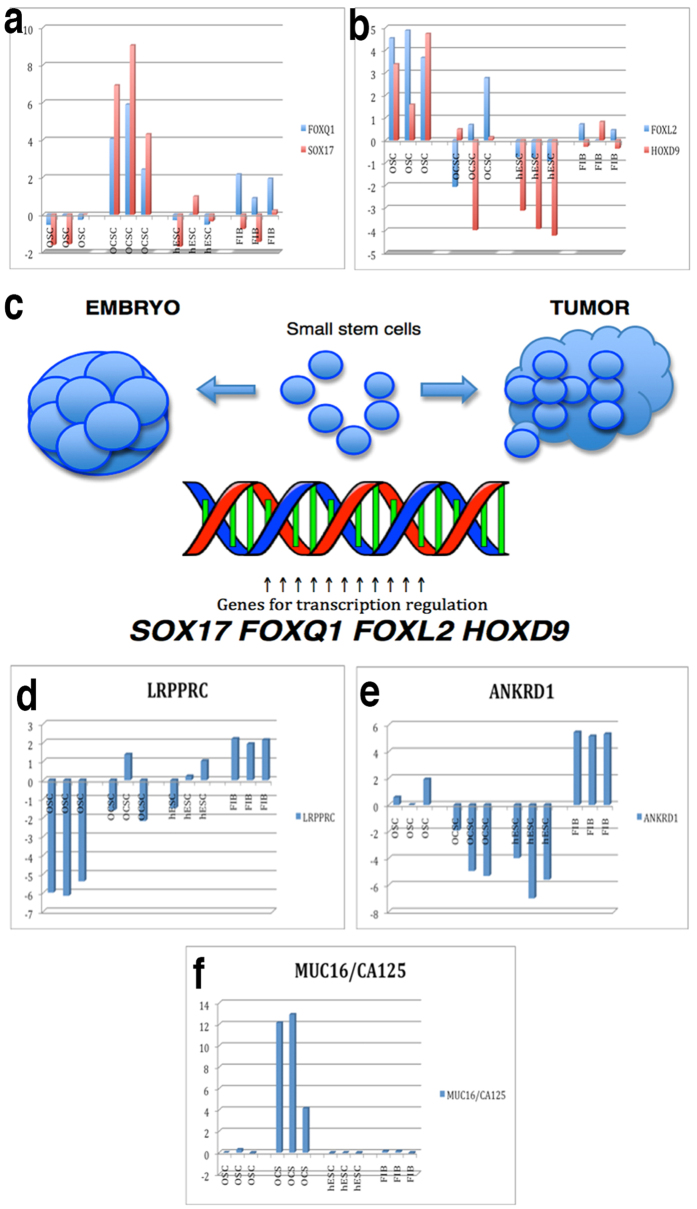
Expression levels of genes that were differentially expressed between the small putative cancer stem cells/tumour-like structures from borderline ovarian cancer and the small stem cells from “healthy” ovaries, including *SOX17*, forkhead box and homeobox genes, which are involved in transcription regulation and biomarker of ovarian cancer MUC16/CA125. (**a**) The *FOXQ1* and *SOX17* genes were highly upregulated in small putative cancer stem cells/tumour-like structures from borderline ovarian cancer (OCSCs) in comparison to small stem cells from “healthy” ovaries (OSCs), human embryonic stem cells (hESCs), and human adult fibroblasts (FIBs). (**b**) The *FOXL2* and *HOXD9* genes were upregulated in small stem cells from “healthy” ovaries (OSCs) in comparison to small putative cancer stem cells from borderline ovarian cancer (OCSCs), hESCs, and fibroblasts (FIBs). The expression of these genes in OCSC samples was more comparable to hESCs than to fibroblasts (FIBs). (**c**) A suggested model of transcription factor encoding *SOX17*, forkhead box, and homeobox genes as determinant factors that direct small ovarian stem cells towards embryogenesis or to the opposite cancerous fate. (**d**) The *LRPPRC* gene was upregulated in small putative cancer stem cells from borderline ovarian cancer (OCSCs) in comparison to small stem cells from “healthy” ovaries (OSCs). Its expression in small putative cancer stem cells was comparable to hESCs but not to human fibroblasts (FIBs). (**e**) The *ANKRD1* gene was downregulated in small putative cancer stem cells (OCSCs) in comparison to “healthy” small stem cells (OSCs), as in hESCs, while human fibroblasts (FIBs) showed a higher expression of this gene. (**f**) In small putative cancer stem cells from borderline ovarian cancer (OCSCs), the expression of the *MUC16*/*CA125* gene, a biomarker of ovarian cancer, was highly overexpressed in comparison to small stem cells from “healthy” ovaries (OSCs), hESCs, and human fibroblasts (FIBs).

We were very interested in comparing the gene expression profiles of small putative cancer stem cells from borderline ovarian cancer (OCSCs) and small stem cells from “healthy” ovaries (OSCs) to elucidate any differences. Therefore, enriched populations of small ovarian stem cells with diameters of up to 5 μm were sorted from cell cultures established from the enzymatically degraded ovarian tissue of three “healthy” women by fluorescence-activated cell sorting (FACS), based on the expression of SSEA-4, as in our previous studies^37^,^39^. All three samples were combined into one sample to efficiently perform FACS sorting of small stem cells. The proportion of SSEA-4-positive cells was relatively low and represented an average of 2.2% of all cells in the ovarian cell cultures upon comparison to the isotype control (Fig. 7a,b). Small putative stem cells were present in the ovarian tissue after enzymatic digestion by collagenase (Fig. 7c) and were concentrated after FACS sorting (Fig. 7d). Small putative stem cells after sorting appeared specifically like small “metal beads”, and their nuclei filled almost the entire cell volumes (Figs 7e,f and 8g,h), as revealed by Hoechst 33258 staining. Both, the ovarian cell cultures established after enzymatic degradation of ovarian tissue and small putative stem cells after sorting were alive, as revealed by propidium iodide (PI) staining (Suppl. Fig. 1). We estimated that an average 94% of cells per ovarian cell culture were alive because they did not stain red when stained by PI (Suppl. Fig. 1a–f). In addition, after sorting, the majority of small putative stem cells were alive, as observed in Fig. 7g–i and Suppl. Fig. 1g–i. The sorted population of cells was divided into three samples for the microarray analysis (OSC1-3).

Furthermore, we manually sorted the tumour-like structures consisting of small putative ovarian cancer stem cells from ovarian cell cultures in three women with borderline ovarian cancer (OCSC1-3). The gene expression profiles of these samples were analysed using microarrays for comparison with three samples of human embryonic stem cells (ESC1-3) and three samples of “somatic” adult human dermal fibroblasts (FIB1-3).

### Genes that were differentially expressed in small putative stem cells from borderline ovarian cancer and “healthy” ovaries after FACS sorting

For the microarray analysis, SuperAmp RNA amplification of the samples was first performed, and Cy3-labelled cDNAs were hybridized to an Agilent Whole Human Genome Oligo Microarray. The differential gene expression analysis (DGA) was performed for OCSCs vs. OSCs to identify differentially expressed transcripts. Functional annotation of transcripts was performed with DAVID Bioinformatics Resources. The microarray experiment description conformed to ArrayExpress with access number E-MEXP-3774.

The analysis revealed that 132 genes were upregulated, and 97 genes were downregulated in the small putative cancer stem cells in comparison to the small stem cells from “healthy” ovaries with high statistical confidence (see Supplemental File 1). These genes belonged to different functional groups related to the regulation of transcription, cell signalling, the cell cycle and apoptosis, secretion, phosphorylation, and cell junction for both upregulated and downregulated genes.

### Regulation of transcription: *SOX17*, forkhead, homeobox and other genes

A prominent group of differentially expressed genes was related to transcription regulation: 13 genes were upregulated (*SOX17*, *ACVR2B*, *CBFA2T2*, *FOXQ1*, *HIVEP3*, *L3MBTL4*, *LRPPRC*, *SIK1*, *SRF*, *TRIM22*, *ZBTB24*, *ZNF148*, and *ZNF721*), and 7 genes (*ANKRD1*, *EPC1*, *FOXL2*, *HOXD9*, *PPARA*, *SIRT4*, and *ZNF30*) were downregulated in the OCSCs compared to the OSCs. These results revealed that *SOX17*, forkhead box (*FOXQ1*, *FOXL2*), and homeobox (*HOXD9*) genes, which are known to regulate cell growth, proliferation, differentiation, embryo development, and the establishment of body axes during embryogenesis, may play important roles in the transition of “healthy” small stem cells to “cancerous” cells (Fig. 8a,b)^44^,^45^,^46^,^47^. As important genes involved in the process of embryogenesis, they may direct development in the opposite – cancerous – direction^46^ when exposed to inappropriate conditions in the body. Therefore, we suggest the transcription factors encoding *SOX17*, forkhead box, and homeobox genes as the factors determining the direction of small ovarian stem cells towards embryogenesis or the opposite cancerous fate (Fig. 8c).

The genes *SOX17* and *FOXQ1* were highly upregulated in small putative cancer stem cells in comparison with “healthy” small ovarian stem cells as well as in hESCs and fibroblasts, as shown in Fig. 8a. In contrast, the genes *FOXL2* and *HOXD9* were significantly downregulated in small putative cancer stem cells in comparison to “healthy” small stem cells; their expression was quite comparable to hESCs but not to fibroblasts (Fig. 8b). Some of the listed transcription factors are expressed in ovarian/tumour tissues and ovarian cancer cell lines and have already been related to various aspects of ovarian cancer, including poor prognosis (*LRPPRC)* and sensitivity to cisplatin (*ANKRD1*), as shown in Table 1^48^,^49^,^50^,^51^,^52^,^53^,^54^,^55^,^56^,^57^,^58^. The *LRPPRC* gene was upregulated (Fig. 8d), and the *ANKRD1* gene (Fig. 8e) was downregulated in small putative cancer stem cells in comparison to “healthy” cells; the expression of both genes was, again, highly comparable to hESCs but not to fibroblasts, as observed in Fig. 8d,e. The protein encoded by *LRPPRC* plays a role in RNA metabolism in both nuclei and mitochondria and is involved in the translation/stability of mitochondrially encoded cytochrome c oxidase (COX) subunits, while protein ANKRD1 is present in the nuclei of endothelial cells and plays a role in endothelial cell activation, according to GeneCards.

### MUC16/CA125 biomarker of ovarian cancer

Among the multitude of interesting genes, the gene MUC16/CA125, which is the most widely used biomarker of ovarian cancer in clinical practice, was highly upregulated in small putative cancer stem cells from “cancerous” ovaries in comparison to cells from “healthy” ovaries, hESCs, and fibroblasts (see Fig. 8f)^59^,^60^.

### Similarity between small putative cancer stem cells and hESCs

From the heatmap analysis, the most expressed genes in small putative cancer stem cells from borderline ovarian cancer were quite comparable to those in hESCs, while small stem cells from “healthy” ovaries and somatic fibroblasts represented completely different populations of cells (Supplemental File 2). Similarly, the least expressed genes in the small putative cancer stem cells were quite comparable to those in the hESCs, while “healthy” small stem cells and somatic fibroblasts represented different populations of cells (Supplemental File 3). These data indicate that the small putative cancer stem cells are more comparable to hESCs than to “healthy” small stem cells and human fibroblasts.

### One-by-one selection of small putative cancer stem cells from borderline ovarian cancer cell culture using a micromanipulation system

Small cells with diameters of 2–4 μm and a known outlook (completely round, slightly yellow, and shining) were one-by-one selected from the borderline ovarian cell cultures (established after enzymatic degradation of ovarian tissue) using a micropipette and a micromanipulation system. The cells were stained by Hoechst, which revealed that cells containing large nuclei that filled almost the entire cell volume (Suppl. Fig. 2).

### Formation of tumour-like structures in organotypic ovarian culture

**Figure 9:**
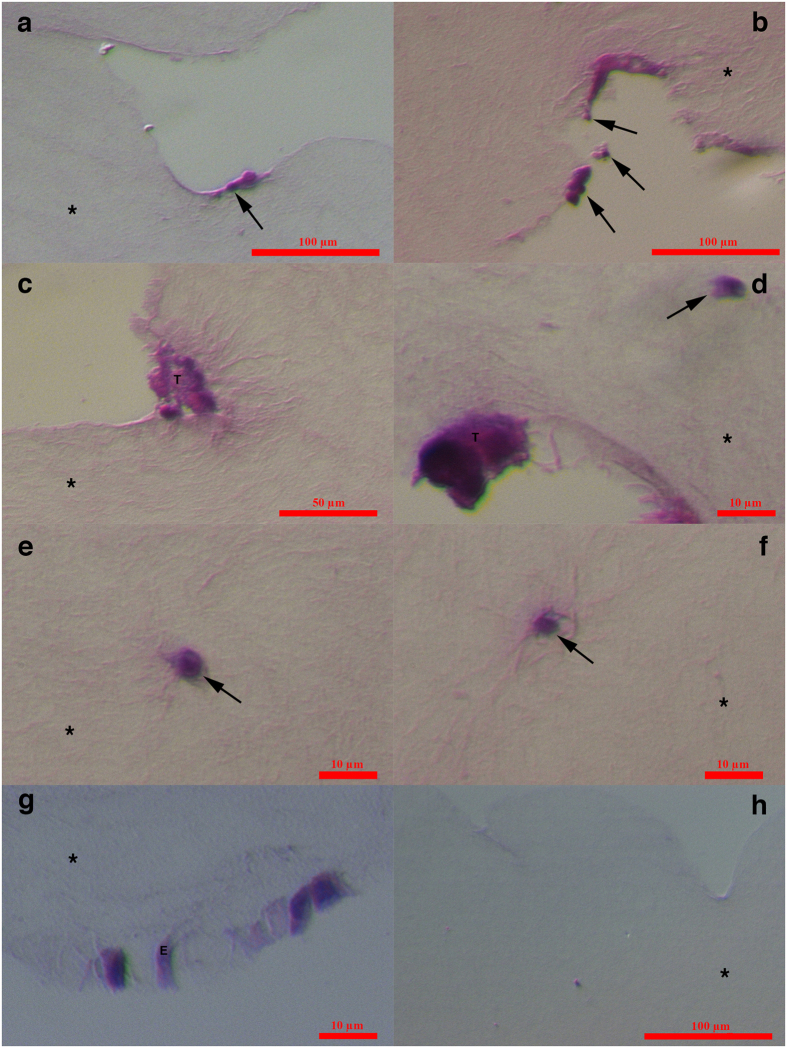
Small putative ovarian cancer stem cells and tumour-like structures in organotypic ovarian culture after haematoxylin and eosin (HE) staining. (**a,b**) Small putative cancer stem cells (arrows) proliferating at the gel surface; (**c**) Tumour-like structure (T) composed of small cells spreading from the gel surface deeper into the gel; (**d**) Tumour-like structure (T), which is possibly mineralized, and a cell invading deeper into the gel (arrow); (**e,f**) Small cells (arrows) with nuclei filling almost the entire cell volume that invaded deeply into the gel; (**g**) Epithelial-like cells (E) forming a layer at the gel surface. (**h**) Gel without the seeding of ovarian cells. Inverted microscope, Hoffman illumination, scale bar, 10 μm (**d–g**), 50 μm (**c**), 100 μm (**a,b,h**). Legend: *-collagen I gel.

A suspension of single cells without tumour-like structures from the ovarian cancer cell culture with a high proliferation of small putative cancer stem cells was cultured for two weeks on collagen I gel to identify how the cells behaved in this microenvironment (see Materials and Methods section). After two weeks, the gel was embedded in paraffin, cut into sections, stained with haematoxylin and eosin (HE) and observed under an inverted microscope (Fig. 9). In some places, it was possible to see groups of small round cells (arrows) with diameters of up to 5 μm and blue stained nuclei filling almost the entire cell volume, which proliferated at the gel surface (Fig. 9a,b). Moreover, the tumour-like structures, which were composed of small cells that invaded from the gel surface deeper into the gel, were identified (Fig. 9c), and some appeared to be mineralized (Fig. 9d). We also observed some single small cells (arrows) that were located deep in the gel (Fig. 9e,f). Among the observed cells, there were also epithelial-like cells, which began to form a layer at the gel surface (Fig. 9g). However, we did not find any comparable cells or structures in gels that were free of the ovarian cell culture (Fig. 9h).

### Small putative cancer stem cells *in situ*

**Figure 10:**
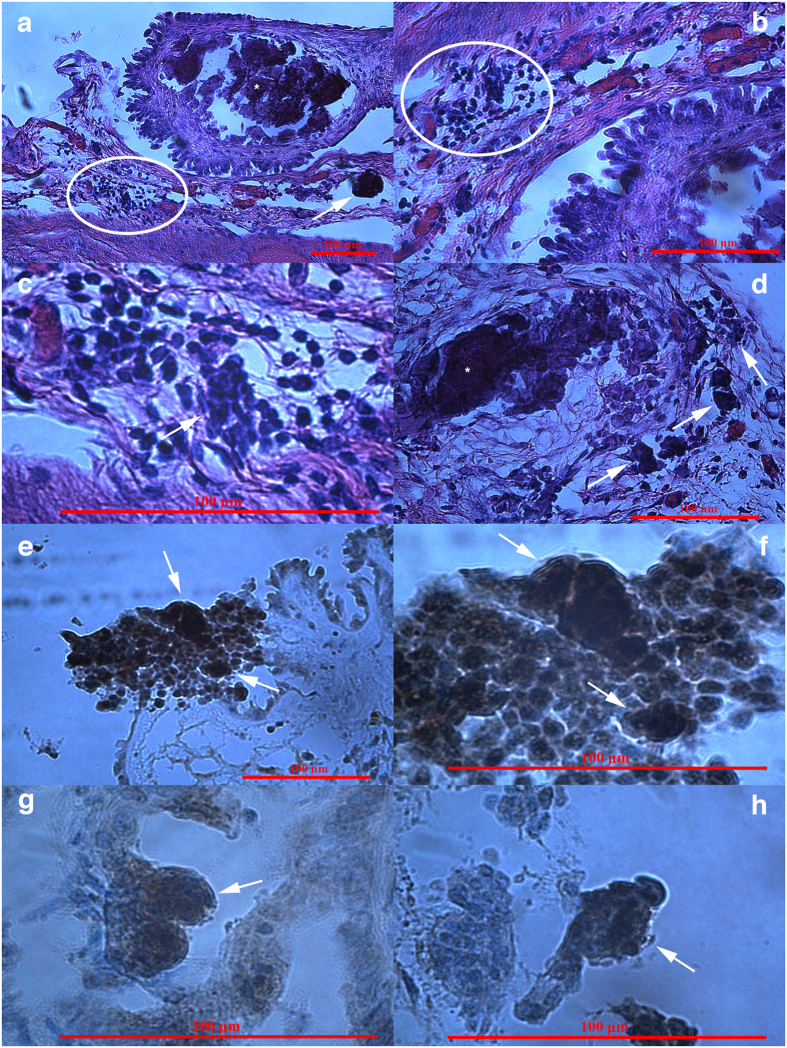
Small putative cancer stem cells forming tumour-like structures after haematoxylin and eosin (HE) staining, positively stained for the pluripotency-related marker NANOG *in situ*, in sections of ovarian tissue from two women with borderline ovarian cancer. (**a**) Proliferating small putative cancer stem cells (circle) that formed tumour-like structures (arrow); a proportion of tumour-like structures were mineralized (asterisks). (**b,c**) Proliferating small putative cancer stem cells (circle) with diameters of up to 5 μm and nuclei filling almost the entire cell volumes and beginning to form a tumour (arrow). (**d**) Proliferating small putative cancer stem cells that formed tumour-like structures (arrows) at two different magnifications. (**e,f**) Small putative cancer stem cells proliferated in the ovarian surface epithelium, formed tumour-like structures (arrows), and stained positively for the pluripotency-related marker NANOG (brown) at two different magnifications. The nuclei of small cells were stained positively for NANOG and filled almost the entire cell volumes. (**g,h**) Different tumour-like structures (arrows) in the layer of ovarian surface epithelium composed of small cells with diameters of up to 5 μm and positively stained for NANOG. Light microscope, scale bar, 100 μm.

We were very interested in finding similar small putative cancer stem cells and tumour-like structures *in situ* in the ovarian sections of the same women with borderline ovarian cancer, provided by our Histopathology Unit. Therefore, we monitored ovarian tissue sections after HE staining based on standard histopathology, and then studied the immunohistochemistry for NANOG, a pluripotency-related marker. For each woman, two slides were observed under a light microscope. We were indeed able to find groups of small putative cancer stem cells in the ovarian tissue of all eight women with borderline ovarian cancer (Fig. 10a–d). These small cells had diameters of up to 5 μm, and the nuclei filled almost the entire cell volumes. At some places, these small cells proliferated in the ovarian surface epithelium, as observed in Fig. 10e,f. They also formed tumour-like structures (Fig. 10e–h). It was possible to clearly see that some tumour-like structures were composed of small round cells (Fig. 10g,h). Moreover, the small round cells and tumour-like structures were positively stained for pluripotency-related marker NANOG (Fig. 10e–h), thus further indicating that the small round cells were indeed putative cancer stem cells.

### Small putative cancer stem cells in other tissues: ovarian teratoma (dermoid) and testicular cancer

#### Ovarian teratoma (dermoid)

**Figure 11:**
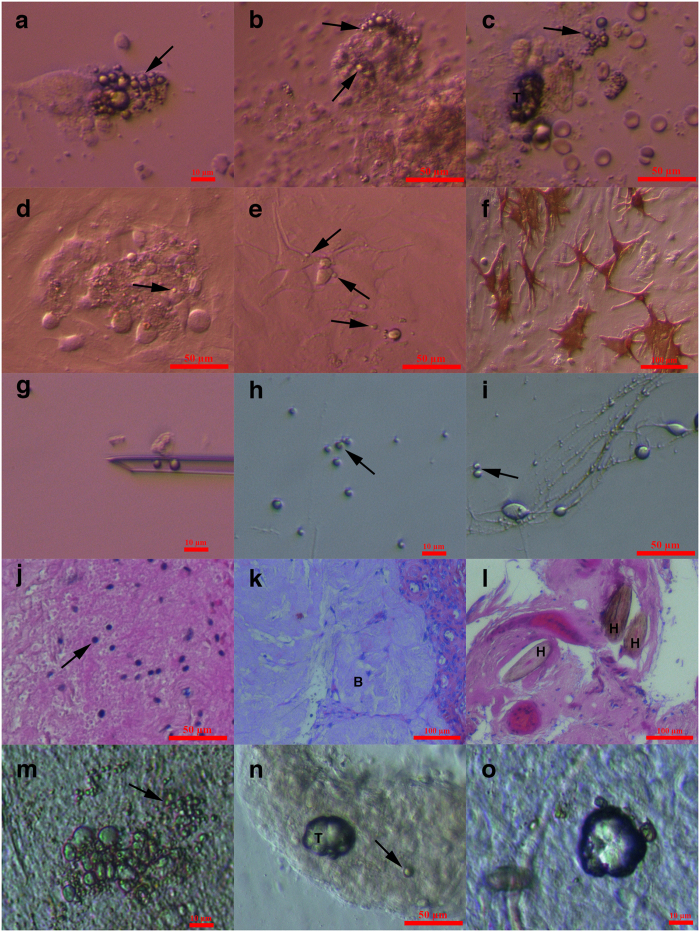
Small putative cancer stem cells with diameters of up to 5 μm in other human tissues *in situ* and *in vitro*: ovarian teratoma (dermoid) in one woman and bilateral testicular cancer in two men. **(a–c)** *In vitro* cell culture of ovarian teratoma (dermoid) tissue after enzymatic digestion with clearly observed small putative cancer stem cells (arrows), which were highly comparable to those from the borderline ovarian cancer and “healthy” ovaries; some cells were growing into bigger cells, and in figure **(c),** small putative cancer stem cells formed a small tumour-like structure (T). **(d–f)** Growth of small putative cancer stem cells (arrows) from an ovarian teratoma into bigger cells **(d,e),** expression of protrusions (**e**,**f**), the development of glial-like cells, which were positively stained for glial-specific marker S100 (red) after exposure of the cell culture to neural differentiation medium (**f**). **(g–i)** Selection of small putative cancer stem cells (arrows) from the ovarian teratoma (dermoid) cell culture using a micromanipulation pipette. After exposure to differentiation medium, small putative cancer stem cells began to grow and developed protrusions **(i). (j)** Small putative cancer stem cells (arrow) *in situ* with diameters of up to 5 μm and nuclei filling almost the entire cell volumes, which were stained blue after HE staining. **(k)** Ovarian teratoma (dermoid) tissue *in situ* with visible brain tissue (B).**(l)** Ovarian teratoma (dermoid) tissue *in situ* with visible hairs (H). **(m–o)** Small putative cancer stem cells (arrows) with diameters of up to 5 μm were found in the testicular cancer tissue just after surgical retrieval. Some of these small putative cancer stem cells grew into larger cells (**m**) or formed tumour-like structures (T) (**n,o**) that were highly comparable to those in the borderline ovarian cancer samples. Tumour-like structures were clearly observed in the testicular tubuli seminiferi (**n**). Scale bar, 10 μm (**a,g,h,m,o**), 50 μm (**b–e,i,j,n**), 100 μm (**f,k,l**).

In addition to borderline ovarian cancer, we enzymatically digested and cultured *in vitro* the ovarian cortex tissue retrieved by biopsy from one patient with ovarian teratoma, dermoid. Interestingly, in this cell culture, we observed several small round cells with diameters of up to 5 μm that were very comparable to the small putative cancer stem cells from borderline ovarian cancer (Fig. 11a–c). These small cells were proliferating, and at some places, they grew into larger cells or formed small tumour-like structures (Fig. 11c), thus representing putative cancer stem cells.

In four wells, the ovarian teratoma cell culture was exposed to neural differentiation medium supplemented with forskolin and basic fibroblast growth factor (FGF) (Fig. 11d–f). In these wells, the small round cells began to grow and then differentiated into glial-like cells, which stained strongly positively (red) for S100, a glial-specific marker, after immunocytochemistry (Fig. 11f), whereas positive staining was not observed in the remaining cell culture that was not exposed to the differentiation medium. This result was further confirmed by the selection and collection of single or groups of small round cells using a micromanipulation pipette (Fig. 11g), which were then cultured in microdroplets of the same neural differentiation medium in paraffin oil (Fig. 11h). The small putative cancer stem cells extensively proliferated, and some grew and formed glial-like cells with several protrusions (Fig. 11i). In addition, similar small round cells were identified *in situ* and in sections of ovarian tissue in the same patient after haematoxylin and eosin staining (Fig. 11j), and the brain tissue was indeed found *in situ* (Fig. 11k) along typical dermoid tissue (Fig. 11l), thus indicating that progenitors were present in the cell culture.

#### Testicular cancer

Additionally, we received testicular biopsies of two patients with aggressive bilateral testicular cancer to cryopreserve the sperm for fertility preservation after orchidectomy. The testicular tissue of both patients was monitored under an inverted microscope just after surgical retrieval to search for sperm. In both patients, spermatogenesis was severely impaired, and there were no spermatozoa present in the tissue. Surprisingly, in the testicular tissue of both patients, there were several small round cells (Fig. 11m) that were highly comparable to the small putative cancer stem cells from the ovaries of the women with borderline ovarian cancer and ovarian teratoma, and in one patient, highly comparable tumour-like structures were found in the testicular tubules (Fig. 11n,o).

## Discussion

In this study, similar populations of small putative stem cells were found in the ovarian surface epithelium/ovarian cortex tissue of women with borderline ovarian cancer and in “healthy” women. In contrast to “healthy” women, however, the small putative cancer stem cells from “cancerous” ovaries intensely proliferated and spontaneously formed tumour-like structures *in vivo*, as observed just after surgical retrieval, and *in situ* and *in vitro* in cell cultures after enzymatic digestion of ovarian cortex tissue. These small putative cancer stem cells and tumour-like structures expressed some markers related to pluripotency and germinal lineage and are proposed to be a novel population of ovarian tumour-initiating stem cells in humans.

The potential involvement of small putative cancer stem cells forming tumour-like structures in the manifestation of ovarian cancer, including borderline ovarian cancer, can be supported by several important facts. First, the character of several genes, which were differentially expressed in the small putative cancer stem cells and “healthy” small stem cells, indicate that they may be involved in the manifestation of ovarian cancer. As reported in the literature, several of these genes have already been related to ovarian cancer when the entire ovarian/tumour tissue or ovarian cancer cell lines were analysed; thus, it cannot be excluded that these ovarian samples contained small putative cancer stem cells, as discovered in this study, when analysed.

A special role is attributed to transcription factors, which are known to be involved in both tumourigenesis and tumour suppression. Among them, *SOX17*, forkhead, and homeobox genes might be crucial and may contribute to the different behaviour of small putative cancer stem cells from borderline ovarian cancer despite their comparable size and morphology to small stem cells from “healthy” ovaries. This seems to be quite rational because all of these genes are related to developmental processes and embryogenesis on one side and to cancer on the other side^44^,^45^,^46^,^47^. The gene *SOX17* is an antagonist of the Wnt signalling pathway, which is related to cancer manifestation, and may contribute to a relatively low aggressiveness of borderline ovarian cancer, although it can lead into high-grade epithelial ovarian cancer when its promoter is methylated^48^. The gene *FOXQ1* is overexpressed in a variety of human cancers, and its upregulation has been associated with poor prognosis in colorectal, breast, and non-small cell lung carcinomas^50^. It has also been reported that *FOXQ1* expression is essential for maintaining cancer cell proliferation, motility, invasion, and epithelial-mesenchymal transition in epithelial ovarian cancer^50^. The transcription factor FOXL2 is extremely important because it is involved in ovarian development and maintenance, folliculogenesis, and differentiation of granulosa cells^61^,^62^,^63^,^64^,^65^. The abnormalities of this gene are related to premature ovarian failure, and the nuclear protein FOXL2 has recently been proposed as a potential novel biomarker for adult-type ovarian granulosa cell tumours in humans^56^,^64^,^65^. Furthermore, it has been found that the homeobox gene family is abnormally expressed in ovarian cancer cell lines, especially *FOXD9*, and disruption of encoding proteins induces apoptosis and retards tumour growth *in vivo*^57^. Overexpression of *HOXD9* in cancer cells augmented the neoplastic phenotype of mucinous ovarian cancer^58^. All these genes might represent novel molecular biomarkers of borderline ovarian cancer, which is still poorly understood at the molecular level, and may represent an important crossroad between embryogenesis and tumour formation in humans.

Second, the gene *MUC16/CA125*, a widely used biomarker of ovarian cancer in clinical practice^59^,^60^, was shown to be highly upregulated in small putative cancer stem cells from borderline ovarian cancer in comparison with small stem cells from “healthy” ovaries, hESCs, and human fibroblasts. Nonetheless, the small putative cancer stem cells were quite comparable to hESCs considering the genes that were the most or least expressed in the two cell types, whereas the expression of these genes was not comparable to “healthy” small stem cells and somatic fibroblasts. It seems that small putative cancer stem cells from borderline ovarian cancer were somehow “reprogrammed” to be more comparable to progenitor hESCs, which are related to tumourigenesis and form teratomas after transplantation into SCID mice^66^.

It is interesting that the majority of tumour-like structures formed by these small putative cancer stem cells mineralized (very possibly calcified) after a period of time, as observed in both the ovarian tissue just after surgical retrieval and in ovarian cell cultures *in vitro*. Also interesting is that ovarian cancer tumours are sometimes calcified, and such calcification tends to occur more commonly in tumours of lower grade^67^. Moreover, our findings may be related to the ultrasound and histopathological observations of atypical ovarian calcifications associated with bilateral borderline ovarian tumours in another study^68^. The mineralization of tumour-like structures formed from small putative cancer stem cells in borderline ovarian cancer may represent an important protective mechanism to prevent the further progression of ovarian tumours and the severity of disease. However, it cannot be excluded that these small putative cancer stem cells and tumour-like structures might be highly aggressive and resistant when this mechanism is absent.

It is becoming clear that different populations of stem cells are involved in the manifestation of ovarian cancer. Small putative cancer stem cells represent one type of stem cell. Our previous studies showed that these small stem cells are present in the ovarian surface epithelium of human adult ovaries in a dormant state^35^,^36^,^37^,^38^,^39^, and it is not excluded that, by their activation and proliferation, they trigger the epithelial-mesenchymal transition and activate subsequent populations of cancer stem cells (e.g., mesenchymal stem cells) that are further involved in ovarian cancer (tumour) invasion. One may also suggest that the surrounding tumour cells may activate the expression of cancer stem cell markers in the germline stem cells, although we did not find any other cell populations that expressed the analysed markers in our cell cultures. Further research is required to offer a firm conclusion.

Small putative cancer stem cells from ovaries resemble a similar population of cells, very small embryonic-like stem cells (VSELs), which were discovered in adult human bone marrow and in peripheral and cord blood by the research group of Ratajczak^69^,^70^. VSELs are proposed to originate in the embryonic epiblast, to persist in human adult organs from the embryonic period of life, to regenerate damaged tissues and to form tumours upon exposure to inappropriate conditions in the body. Our observation may strongly support this idea.

In this study, a very similar population of small putative cancer stem cells and tumour-like structures were also identified in ovarian teratoma, dermoid, and testicular cancer tissue, thus indicating that these small putative stem cells might persist from the embryonic period of life, namely from indifferent gonads, and could be widely implicated in cancer manifestation in both the ovaries and testicles. This novel population of tumour-initiating stem cells related to borderline ovarian cancer deserves to be further studied to better understand the manifestation of ovarian cancer and to offer more successful diagnoses and treatments in the future.

## Material and Methods

### Patients

This study was approved by the Slovenian Medical Ethical Committee (Ministry of Health of the Republic of Slovenia, No. 135/09/09 and 154/07/10) considering research using stem cells from human adult ovaries and is in compliance with the Helsinki Declaration. Ovarian tissue from eight women with a mean age of 46.4 (min. 23–max. 66) years, diagnosed with borderline ovarian cancer, was included in this study along with tissue from three “healthy” women without cancer. The ovarian tissue from one “healthy” woman was surgically retrieved to better perform a histological diagnosis of ovarian insufficiency (premature ovarian failure) in terms of potential follicles in the ovarian cortex. For the two remaining “healthy” women, the ovaries were surgically removed to prevent breast cancer, according to the recommendation of an oncologist. Breast cancer is in the familiar anamnesis of both patients, and they are both suffering breast cysts and mutation of the *BRCA1* gene. The histopathologists did not find any malignancy in the retrieved ovarian tissue. The ovarian tissue was included in the study after written informed consent of the women who decided to voluntary donate a part of their ovarian tissue for research along with routine histopathological diagnosis of their disease.

Table 2 provides the list of all patients with borderline ovarian cancer and “healthy” women who donated their ovarian tissue for this study according to the initials of their names and their year of their birth; all patients are indicated in the figures of cell cultures, immunostaining, DAPI or Hoechst 33258 staining, electron microscopy and microarray analysis.

### Ovarian tissue processing

Ovarian tissue biopsies were enzymatically digested in two enzyme solutions. After mincing the biopsies using a scalpel, they were incubated for 15 minutes in 0.7 mg/ml collagenase type XI (Sigma-Aldrich) at 37 °C and centrifuged for 8 minutes at 1400 rpm. The supernatant was then discarded, the pellet resuspended in a 1:1 mixture of hyaluronidase (SynVitro Hydase, Origio) and 0.7 mg/ml collagenase type XI. After 15 minutes of incubation at 37 °C, 10% of foetal bovine serum (FBS, Gibco) was added, and the sample was centrifuged for 8 minutes at 1400 rpm. The supernatant was then discarded, and the cells were washed with basal medium consisting of DMEM/F-12 culture medium (Sigma-Aldrich) with 3.7 g/l NaHCO_3_ and 1% penicillin/streptomycin (Sigma-Aldrich) with the pH adjusted to 7.4 using 1 M NaOH. After washing, the cells were resuspended in DMEM/F12 with 20% FBS and passed through a 70-μm cell strainer (BD Falcon). These cells were then seeded into 12-well culture plates precoated with 0.2% gelatine. The culture medium (DMEM/F12 with 20% of FBS) was changed every 3 days, and the cells were passaged when needed using 0.15% trypsin-EDTA (Sigma-Aldrich).

### Origin and culture of human embryonic stem cells (hESCs)

hESCs (H1 cell line, WiCell, WI, USA) were cultured on MEFs in hESC medium, which is generally used to culture human embryonic stem cells. The medium was composed of DMEM/F12 (Sigma-Aldrich), 20% KnockOut Serum Replacement (Gibco), 1 mM L-glutamine (PAA), 1% non-essential amino acids (PAA), 0.1 mM 2-mercaptoethanol (Invitrogen), 13 mM HEPES (Gibco) and 8 ng/ml human basic FGF (Sigma-Aldrich). To collect hESCs for the microarray analysis, the hESC cell cultures were first treated with collagenase type IV (Sigma-Aldrich) to remove MEFs, and then the hESC colonies were scraped from culture plates using a cell scraper. The scraped hESCs were centrifuged for 5 minutes at 1000 rpm and washed once with PBS. Washed hESCs were lysed using SuperAmp™ Lysis Buffer and stored at −20 °C until further use.

### Origin and culture of human fibroblasts

Human adult dermal fibroblasts were provided by Cascade Biologics (Invitrogen) and were cultured in DMEM/F12 (Sigma-Aldrich), supplemented with 10% FBS (Gibco). To collect cells for the microarray analysis, the fibroblast cell cultures were washed with PBS and incubated in 0.15% trypsin-EDTA (Sigma-Aldrich) for 3 minutes to detach the fibroblasts from the cell plates; the detached fibroblasts were centrifuged for 6 minutes at 1200 rpm and washed once with PBS. The washed fibroblasts were lysed using SuperAmp™ Lysis Buffer and stored at −20 °C until further use.

### Time-lapse microscopy of ovarian cell cultures

Ovarian cell cultures were monitored using a PrimoVision^TM^ (VitroLife, Sweden) time-lapse camera that was installed inside a CO_2_-incubator and connected to an exterior computer running PrimoVision software to set up and monitor the cells. All data were recorded and stored. Tumour-like structures and the surrounding cells were manually aspirated from the ovarian cell cultures using a pipette and transferred to microdroplets of culture medium in microwells under paraffin oil for monitoring by the time-lapse camera. Each sample (tumour-like structures with the surrounding cells) was monitored for at least two months.

### Immunocytochemistry of ovarian cell cultures

The cells were immunocytochemically stained using two approaches, employing either biotin-conjugated or fluorophore-conjugated secondary antibodies.

#### DAB procedure

For use of the biotin-conjugated secondary antibodies, the cells were fixed using 4% paraformaldehyde (PFA) for 10 minutes, permeabilized with 0.2% Triton for 10 minutes (when intracellular protein was analysed), incubated with 3% H_2_O_2_ for 20 minutes and then in 10% FBS for 20 minutes. The cells were then incubated for 1 hour in primary antibodies (mouse anti-SOX-2 monoclonal antibodies, diluted 1:100, Millipore; rabbit anti-DDX4 (VASA) polyclonal antibodies, diluted 1:100, Millipore; mouse anti-STELLA (DPPA3) monoclonal antibodies, diluted 1:200; Millipore; rabbit anti-S100 polyclonal antibodies, diluted 1:500, DakoCytomation) and then for 30 minutes in secondary antibodies. For the secondary antibodies, biotinylated polyclonal rabbit anti-mouse immunoglobulins (diluted 1:400, DakoCytomation) or biotinylated polyclonal goat anti-rabbit immunoglobulins (diluted 1:600, DakoCytomation) were used. After thorough washing, the cells were incubated for 20 minutes with ABC reagent (Vectastain Elite ABC Kit, Vector Laboratories) and then in diaminobenzidine substrate (Sigma-Aldrich) until brown staining appeared. Stained cells were observed under an inverted microscope (Hoffman illumination).

#### Immunofluorescence

When using fluorophore-conjugated secondary antibodies, the cells were fixed in 4% PFA for 10 minutes, permeabilized with 0.2% Triton for 10 minutes (when intracellular protein was analysed) and then in 10% FBS for 20 minutes. Then, the cells were incubated in primary antibodies (rabbit anti-OCT4 polyclonal antibodies, diluted 1:100, Stemgent; mouse anti-NANOG monoclonal antibodies, diluted 1:50, Abgent; mouse anti-SSEA-4 monoclonal antibodies, diluted 1:100, Chemicon; mouse anti-cytokeratin monoclonal antibodies, diluted 1:200, Miltenyi) and after 1 hour, the cells were incubated in secondary antibodies (anti-mouse immunoglobulins conjugated to Alexa Fluor 488, diluted 1:200, and anti-rabbit immunoglobulins conjugated to Cy3, diluted 1:200, both of Molecular Probes) for 30 minutes at room temperature. After washing, the cells were counterstained with 4,6-diamidino-2-phenylindole (DAPI) to stain the nuclei. Stained cells were observed under a Nikon fluorescence microscope. Positive staining for NANOG, SSEA-4 and cytokeratin was observed as green staining, and positive staining for OCT4 was observed as red staining.

As a negative control, cells were treated in the same way as described above (samples) except the step when the primary antibodies are added to cells was modified. In negative control cells, the primary antibodies were not added, and the cells were exposed only to 1% FBS in PBS for the same time period that the experimental cells (samples) were incubated with the primary antibodies. The negative controls were exposed to 1% FBS in PBS because the antibodies were diluted in this solution when used for staining.

### Neural differentiation of cell cultures

To induce neural differentiation, the cells were cultured in DMEM/F12 medium supplemented with 2% FBS, 80 ng/ml human basic fibroblast growth factor (Sigma-Aldrich), 30 μM forskolin (Sigma-Aldrich), 1% non-essential amino acids (Gibco), 0.1 mM 2-mercaptoethanol (Gibco) and 1% insulin-transferrin-selenium (Gibco). After 2 weeks of culture, the cell cultures were analysed for the expression of glial marker S100 using immunocytochemistry according to the procedure described above.

### Fluorescence-activated cells sorting (FACS) based on SSEA-4 expression

Cells from one twelve-well plate of each culture were trypsinized (0.15% solution of trypsin-EDTA), combined, and sorted based on SSEA-4 expression. The SSEA-4-positive cells were isolated from a cell suspension of the combined sample using fluorescence-activated cell sorting (FACSAria, BD Biosciences, San Jose, CA, USA). Briefly, 10^6^ cells were resuspended in PBS with 1% penicillin/streptomycin (Sigma Aldrich) and stained with anti-SSEA-4 antibodies (clone MC813-70; BD Pharmingen, San Jose, CA, USA) conjugated with phycoerythrin (PE). A sample stained with an appropriate isotype control (PE Mouse IgG3, κ; BD Pharmingen) was examined in parallel. All antibodies were added at saturation concentrations, and the cells were incubated for 30 minutes in the dark, then washed and resuspended for sorting in PBS with penicillin/streptomycin at a concentration of 2 × 10^6^ cells/ml. Next, FACS cells were cultured in supplemented DMEM/F-12 culture medium with 20% foetal bovine serum. Droplets of sorted cells were stained with DAPI (VECTASHIELD Mounting Medium for fluorescence, Vector Laboratories) or Hoechst 33258 and monitored under a fluorescence microscope. For Hoechst staining, the selected cells were transferred in a 10 μg/ml solution of bisbenzimide (Hoechst 33258, Sigma-Aldrich) and incubated at room temperature in the dark. After 10 minutes, the stained cells were observed under fluorescence microscope.

### Gene expression analyses by microarrays

#### Microarrays

Ovarian cell samples were lysed using SuperAmp™ Lysis Buffer and stored at −20 °C. When collected, the samples were analysed by Miltenyi Biotec Microarray Service (Bergisch Gladbach, Germany) according to established protocols. First, the SuperAmp RNA amplification was performed. Amplified cDNA samples were quantified using an ND-1000 spectrophotometer (NanoDrop Technologies, Wilmington, DE, USA). The integrity of the cDNA was checked using an Agilent 2100 Bioanalyzer platform (Agilent Technologies, Wokingham, UK) (Suppl. Fig. 3). The Cy3-labeled cDNAs were hybridized overnight to an Agilent Whole Human Genome Oligo Microarray 8 × 60 K and washed. Fluorescence signals of the hybridized Agilent microarrays were detected using Agilent’s Microarray Scanner System (Agilent Technologies). Agilent Feature Extraction Software was used to read out and process the microarray image files.

#### Statistical analysis of microarray data

The biostatistical analysis was performed by Miltenyi Biotec Microarray Service (Bergisch Gladbach, Germany). Differential gene expression analysis (DGAs) was performed on ovarian cancer stem cells (OCSCs) and ovarian stem cells (OSCs) to identify differentially expressed transcripts. To determine the differential gene expression-derived output data, the files were further analysed using the Rosetta Resolver gene expression data analysis system (Rosetta Biosoftware). The data were preprocessed by normalization and correlation analysis.

Data preprocessing covers all necessary data transformations, an assessment of variability between the samples, and the detection of potential outliers by a global correlation analysis. After background correction, quantile normalization was conducted between arrays. The effect of the normalization procedures on the overall distribution of the signal intensity values is demonstrated in the boxplot image of Supplemental Fig. 4. The boxplot graphs represent the distribution of the interquartile range of the data points within the boxes for each sample. The median is represented by a black horizontal line. The range of the whiskers corresponds to 1.5-fold of the interquartiles, and outliers are displayed as circles. The boxplots of the unadjusted log2 signal intensity values show differences in the scales of the intensities. The ranges of the intensity values were adjusted to the same scale upon normalization – the median values were all at similar levels, and the total distributions of the log2 normalized signal intensity values were almost identical. Finally, the normalized intensities were log2-transformed and served as the basis for further analyses.

Heatmaps were created based on the ratio data in the log2 space to obtain a more intuitive visual inspection of the gene expression data. Student’s t-tests were performed on each gene separately using the normalized log2 intensity data. Statistical significance was set at p < 0.05. The genes selected as reliable candidates for differentially expressed genes were required to show at least a 16-fold average expression difference (log ratio = 4) between the sample groups. Due to some variability, a more stringent selection was performed for individual comparisons to obtain a statistically high confidence list of differentially expressed genes. To elucidate the gene functions and pathways, DAVID Bioinformatics Resources 6.7 was used. We focused on genes differentially expressed between OCSCs and OSCs.

### Manual isolation of small putative ovarian cancer stem cells from borderline ovarian cancer cell culture

The borderline ovarian cancer cell culture was first treated with 0.15% trypsin-EDTA to detach the cells from the culture plate and to achieve a single cell suspension. The suspension of cells was then centrifuged for 6 minutes at 1200 rpm, washed once with PBS and then resuspended in PBS. To enable the manual selection of cells using a hydraulic micromanipulation system (Narishige), the suspension of cells was transferred to the lids of Falcon 35 mm cell culture dishes in 50 μm droplets and covered with liquid paraffin to prevent evaporation of the PBS. The cell suspension droplets were then inspected under a heat-stage inverted microscope (with a digital camera connected to the microscope) to identify small round cells with diameters of 2–4 μm. When such a cell was found, it was transferred to the fresh droplet (10 μm) of PBS located on another 35 mm cell culture dish lid and covered with liquid paraffin. The small cells were transferred one-by-one using a micropipette (diameter 11 μm; Hatching Pipette, 60 mm, 30° angle, Swemed by Vitrolife), which was installed on a micromanipulation system. To stain the manually selected cells with Hoechst 33258, the cells were transferred with a transfer pipette (Swemed by Vitrolife) to a solution of Hoechst 33258 (10 μg/ml) shortly before placing the cells on a microscopic slide with cavities (Marienfeld). After 15 minutes of incubation, the cells were observed under a fluorescence microscope, and the cell nuclei were stained blue.

### Propidium iodide (PI) staining

The cell culture was first treated with 0.15% trypsin-EDTA to detach the cells from the culture plate and to achieve a single cell suspension. The suspension of cells was then centrifuged for 6 minutes at 1200 rpm, washed once with PBS and then resuspended in PBS with Hoechst 33258 (5 μg/ml) and propidium iodide (1 μg/ml). After 15 minutes, the cells were centrifuged for 6 minutes at 1200 rpm, resuspended in PBS and observed under a fluorescence microscope. The non-viable cells expressed red fluorescence, while the vital cells were not stained red. The proportion of viable cells was estimated (per 200 cells).

### Organotypic ovarian cell culture

The cell culture with highly proliferated small putative cancer stem cells was cultured on collagen I gel to the behaviour of the cells in a 3D microenvironment. The gel was prepared as follows: Working on ice to cool all reagents to 4 °C, the collagen I solution (Gibco) was diluted from a starting concentration of 5 mg/ml to 4 mg/ml using appropriate volumes of 10x DMEM/F12, FBS and NaOH. This solution was then placed in a Millicell cell culture insert (with a diameter of 12 mm and a pore size of 0.4 μm; Merck Millipore) and incubated at 37 °C until a firm gel was formed. After 1 hour, the gel was rinsed with 1xDMEM/F12 and seeded with cells. The cells were derived from ovarian cancer cell culture, which was treated with 0.15% trypsin-EDTA to detach the cells from the plastic surface and to retrieve a single cell suspension. The suspension of cells was then centrifuged for 6 minutes at 1200 rpm, washed with DMEM/F12 and resuspended in DMEM/F12 with 20% FBS. Approx. 10,000 cells were seeded on the gel from this suspension. The cell culture insert was then placed in a well of a 4-well cell culture plate, and DMEM/F12 with 20% FBS was added to a well around the cell culture insert to feed the cells and to prevent drying out of the gel. The culture media was changed every 3 days, and after 2 weeks, the gel with cells was removed from the cell culture insert, washed with PBS, fixed and embedded in a paraffin block. Consecutively, 3–5 μm sections were cut, placed on silane-coated slides (Menzel-Glaser Superfrost) and dried at 60 °C in a dryer for one hour. After deparaffinization and rehydration, the sections were stained with haematoxylin and eosin (HE), which stained the cell nuclei blue.

### Immunohistochemistry of ovarian tissue sections for NANOG

The analysis was performed on a tissue microarray (TMA) of tissue samples from women with ovarian borderline cancer. Along with haematoxylin and eosin (HE) staining, sections of ovarian tissue were stained on NANOG. The ovarian/tumour tissue was transplanted to paraffin blocks. Consecutively, 3–5 μm sections were cut from each block. The paraffin sections were placed on silane-coated slides (Menzel-Glaser Superfrost) and dried at 60 °C in a dryer for one hour. The following immunohistochemical staining was performed in an automatic slide stainer (Ventana BenchMark GX). After deparaffinization, antigen retrieval (HIER) was performed with CC1 Ventana reagent at pH 7–8 for 48 minutes. This was followed by hand application of the primary antibody. The slides were incubated for 30 minutes with rabbit anti-human NANOG monoclonal antibody (ab109250, Abcam, Cambridge, MA, USA, dilution 1:25) at 37 °C. Then, Ventana OptiView Kit was used to detect the antigen. The elimination of the primary antibody served as a negative control. In each woman, up to two ovarian sections were stained. The stained slides were monitored under a light microscope (at magnifications up to 1000×). After HE staining, the cell nuclei were stained blue, and NANOG-positivity was expressed by brown colour. We focused on nuclear NANOG staining, which matched the blue HE staining.

## Additional Information

**How to cite this article**: Virant-Klun, I. and Stimpfel, M. Novel population of small tumour-initiating stem cells in the ovaries of women with borderline ovarian cancer. *Sci. Rep.* **6**, 34730; doi: 10.1038/srep34730 (2016).

## Supplementary Material

Supplementary Information

Supplementary Video 1

Supplementary Video 2

Supplementary File 1

Supplementary File 2

Supplementary File 3

## Figures and Tables

**Table 1:** Relations among transcription factors; upregulated or downregulated transcription factors in small putative cancer stem cells from borderline ovarian cancer in comparison to those from “healthy” ovaries and to ovarian cancer reported in the literature^48^,^49^,^50^,^51^,^52^,^53^,^54^,^55^,^56^,^57^,^58^.

Gene (transcription factor)	OVARIAN CANCER			
	Related to ovarian cancer	Type of ovarian cancer	Related to prognosis of ovarian cancer	Reference
Upregulated				
***SOX17***	**+**	High-grade epithelial	**−**	48
***ACVR2B***	**+**	**−**	**−**	49
***CBFA2T2***	**−**	**−**	**−**	**−**
***FOXQ1***	**+**	Epithelial	**−**	50
***HIVEP3***	**−**	**−**	**−**	**−**
***L3MBTL4***	**−**	**−**	**−**	**−**
***LRPPRC***	**+**	**−**	**+**	51
***SIK1***	**−**	**−**	**−**	**−**
***SRF***	**+**	Epithelial, serous, endometrioid	**−**	52, 53
***TRIM22***	**+**	**−**	**−**	54
***ZBTB24***	**−**	**−**	**−**	**−**
***ZNF148***	**−**	**−**	**−**	**−**
***ZNF721***	**−**	**−**	**−**	**−**
Downregulated				
***ANKRD1***	**+**	Epithelial	**+**	55
***EPC1***	**−**	**−**	**−**	**−**
***FOXL2***	**+**	Granulosa	**−**	56
***HOXD9***	**+**	Epithelial, mucinous	**−**	57, 58
***PPARA***	**−**	**−**	**−**	**−**
***SIRT4***	**−**	**−**	**−**	**−**
***ZNF30***	**−**	**−**	**−**	**−**

**Table 2:** The data of the patients included in this study: eight patients with borderline ovarian cancer and three “healthy” patients without cancer.

Name/initials of a patient (number of culture)	Year of birth	Figure
Women with borderline ovarian cancer		
K. M. (**Culture 20**)	1961	Figures 3b–d,e,o and 4a,b,d,e,f–o
C. D. (**Culture 24**)*	1956	Figures 2a,b,d,f, 3f–j,m,n and 9a–d
K. B. (**Culture 25**)*	1978	Figure 2h–l
B. A. (**Culture 26**)*	1988	Figure 5d–i, m–o;
Ž. J. (**Culture 29**)	1977	Figures 2c,e,g,m–o, 3k, 4c, 5a–c,j–l and 6
V. B. (**Culture 30**)	1971	Figure 3l
O. A. (**Culture 51**)	1925	Figure 7g,h and Supplemental Figure 1a–l
B. M. (**Culture 54**)	1947	Figure 1a–h
V. A. (**Culture 58**)	1962	Figure 9e–h
Healthy women (no cancer)		
P. P. A. (**Culture 14**)*	1975	Figure 3a
P. R. A. (**Culture 16**)*	1972	Figure 7c,e,f
B. D. (Culture 23)*	1962	Figure 7d

^*^Microarray analysis.
