# Supplementary material for: Novel population of small tumour-initiating stem cells in the ovaries of women with borderline ovarian cancer
Source: Sci Rep. 2016 Oct 5;6:34730. doi: 10.1038/srep34730 (PMC5050448; doi:10.1038/srep34730)
Supplement: Supplementary Information [file srep34730-s1.doc]

**Novel population of small tumour-initiating stem cells in the ovaries of women with borderline ovarian cancer**

Irma Virant-Klun and Martin Stimpfel

**Supplementary information**

**Supplementary Video 1**: Time-lapse monitoring of tumour-like structure from borderline ovarian cancer: small putative cancer stem cells proliferate at the surface of the tumour and fuse with it to grow.

**Supplementary Video 2**: Time-lapse monitoring of ovarian tissue with tumour-like structures from borderline ovarian cancer: small putative cancer stem cells proliferate at several places, form “chains”, and in some places form small tumour-like structures de novo.

**Supplementary Figure 1**: **Evaluation of ovarian cell viability using propidium iodide (PI) and Hoechst 33258 staining.**

(a-f) The majority (on average 94 %) of cells in ovarian cell cultures before sorting were viable and did not stain red by propidium iodide. (g-l) The majority of small putative stem cells after sorting were viable. The blue (Hoechst) stained nuclei filled almost the entire cell volumes and cells did not stain red by propidium iodide staining. Fluorescence microscope (a-i,k,l), light microscope (j). Scale bar, 10μm (g-i), 100μm (a-f,j-l). Legend: *-air bubble (was of significantly different appearance than small stem cells and did not stained by Hoechst).


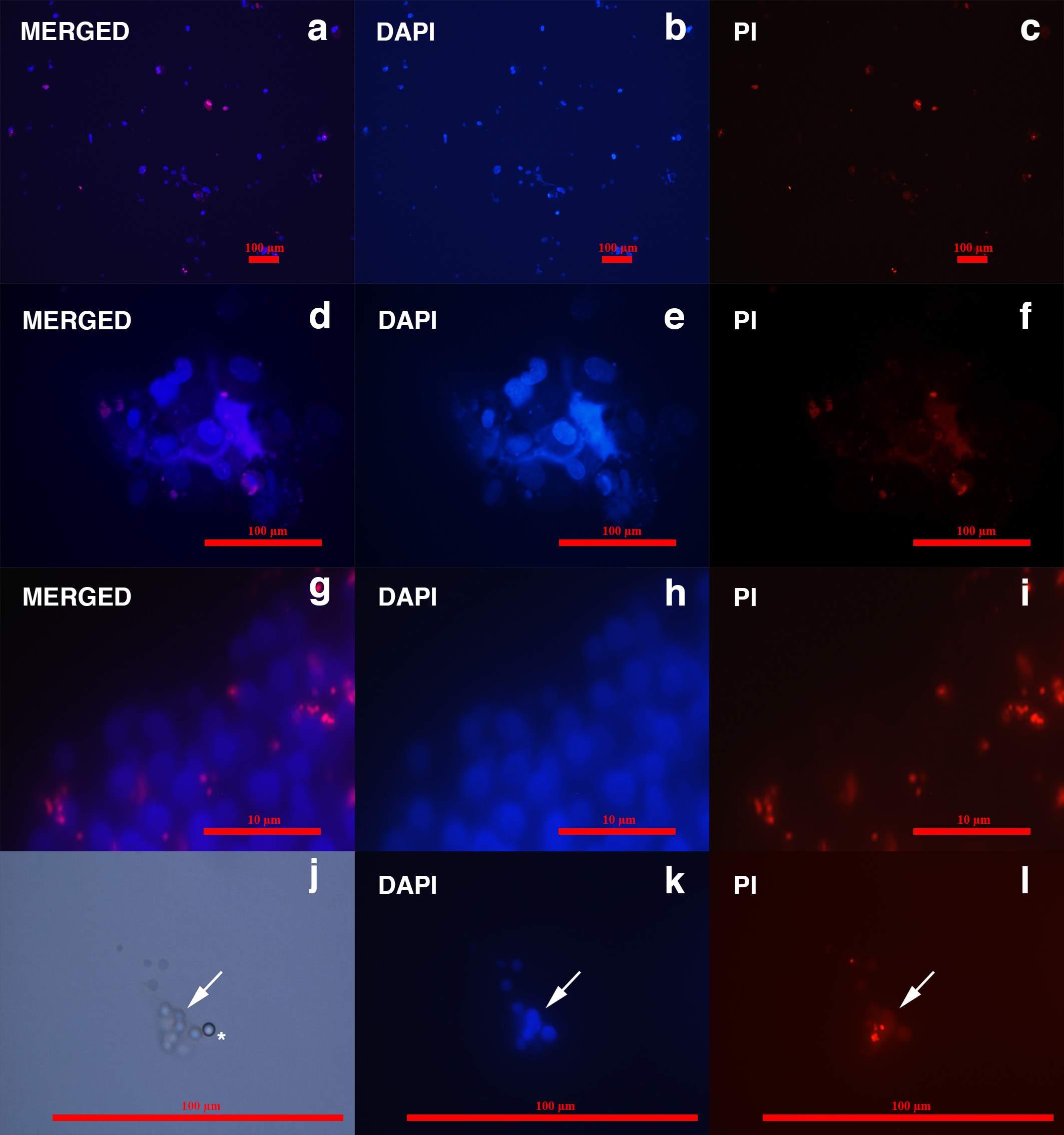


**Supplementary Figure 2:** **Hoechst 33258 staining of small putative cancer stem cells with diameters of up to 5 μm manually isolated from cell culture in one patient with borderline ovarian cancer using a micromanipulation system.**

(a,b) Isolation of small putative cancer stem cells by micropipette (diameter 11 μm; Hatching Pipette, 60 mm, 30^o^ angle, Swemed by Vitrolife, Sweden, ref. 14324); (c-f,i,j) Small putative cancer stem cells attached to cell debris; (g,h,k,l) Single putative cancer stem cells; (d-l) Nuclei of small putative cancer stem cells stained blue after DAPI staining, while cell debris did not stain (d,f,j). Legend: (d,f,h,j,l) fluorescence microscope, (a-c,e,g,i,k) light microscope. Scale bar, 10μm (a,b,), 100μm (c-l).


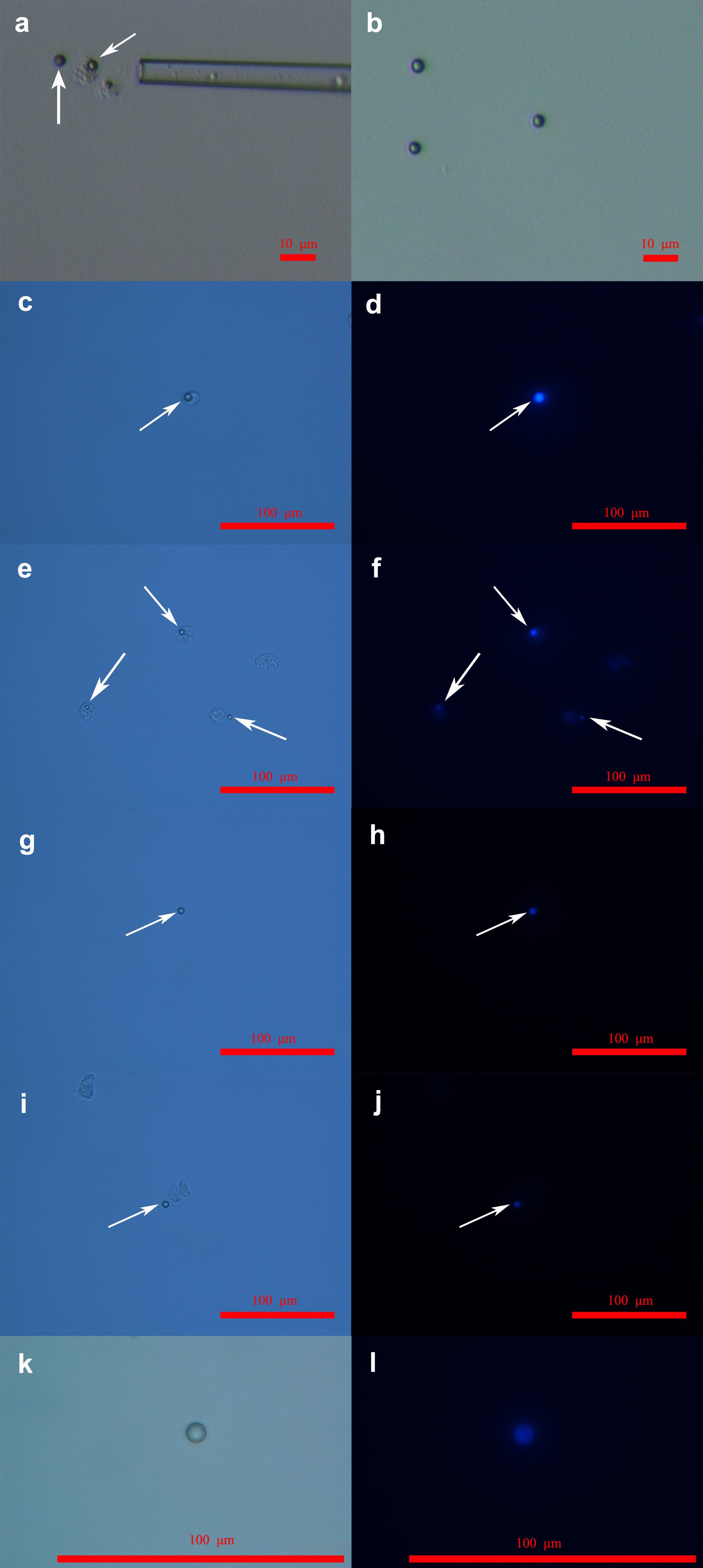

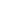


**Supplementary Figure 3: Gel image (A) and electropherogram (B) of amplified cDNA samples. As a reference, the DNA molecular weight ladder (in nucleotides, nt) is shown in the first lane. The lowest migrating, green band represents an internal standard. Scaling of the y-axis is done automatically, relative to the strongest signal within a single run.**


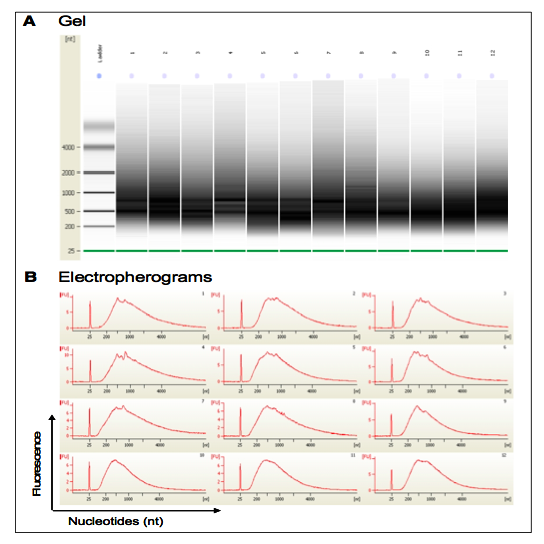


**
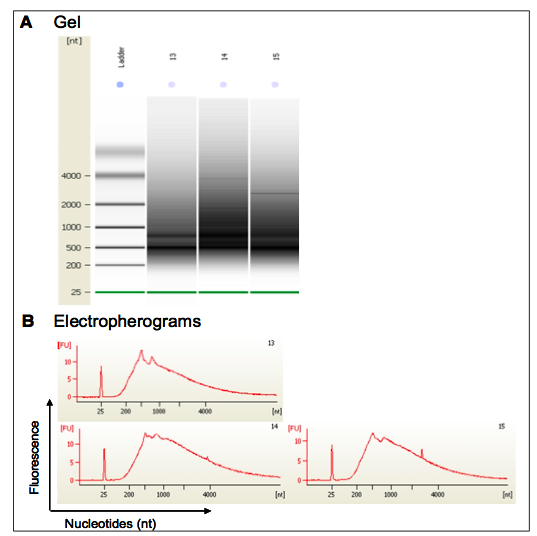
**

**Supplementary Figure 4: Boxplot graphs showing the distribution of the log2 intensity data before (a) and after (b) quantile normalization.**

**
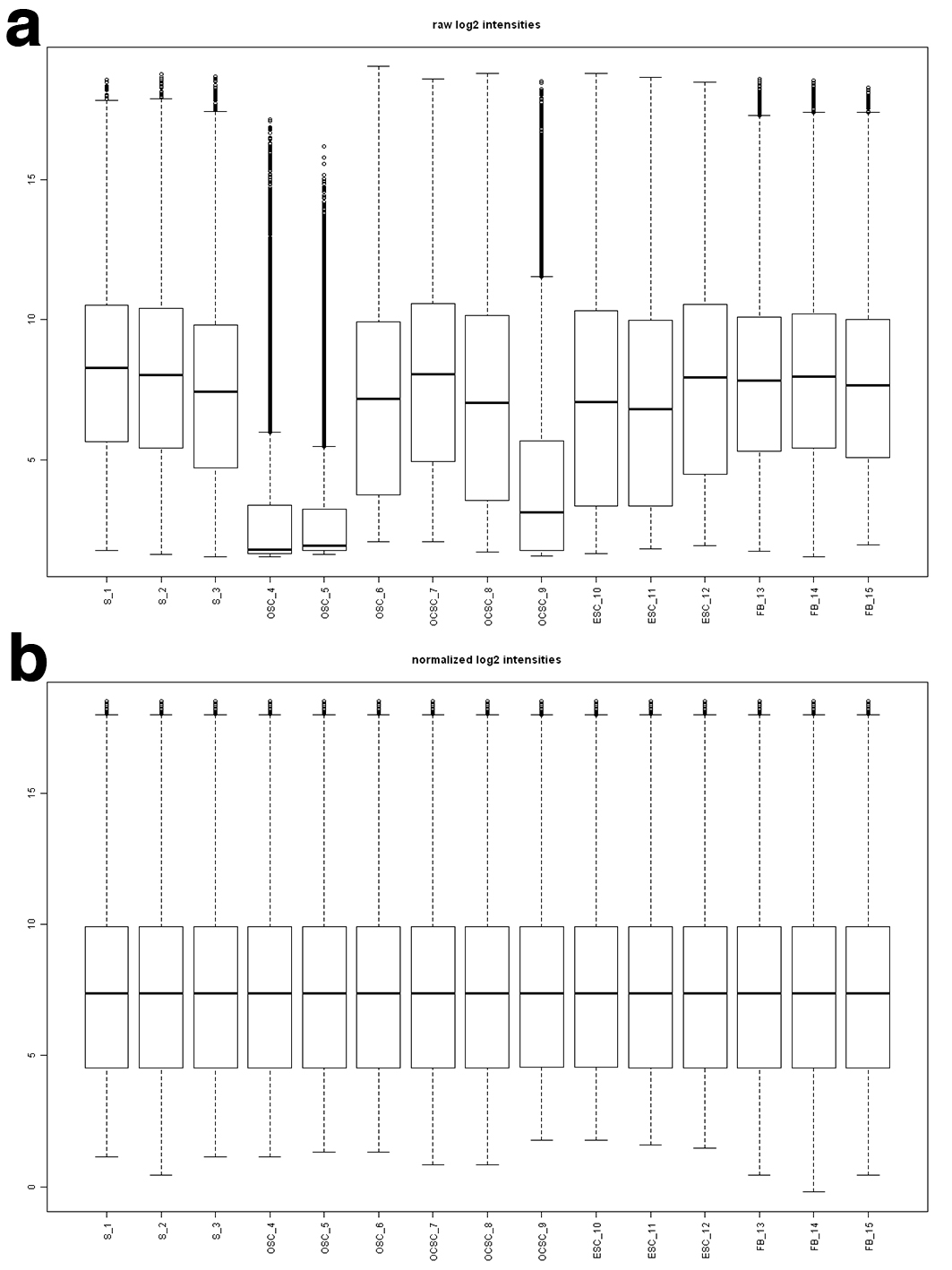
**

**Supplementary File 1**: DGA comparison of microarray data between small putative cancer stem cells from borderline ovarian cancer (OCSCs) and small stem cells from “healthy” ovaries (OSCs) which revealed 132 upregulated and 97 downregulated genes in small putative cancer stem cells at a confident statistical significance.

**Supplementary File 2**: Heatmap for the most expressed genes in small putative cancer stem cells from borderline ovarian cancer (OCSC_7, OCSC_8, OCSC_9) in comparison with other groups of cells: small stem cells from “healthy” ovaries (OSC_4, OSC_5, OSC_6), human embryonic stem cells (ESC_10, ESC_11, ESC_12), fibroblasts (FB_13, FB_14, FB_15), and the whole ovarian cell culture from “healthy” ovaries without sorting the small putative stem cells (S_1, S_2, S_3). OCSCs were quite comparable to ESCs for a higher group of these genes, while they completely differ from other types of cells.

**Supplementary File 3**: Heatmap for genes which were least expressed in small putative cancer stem cells from borderline ovarian cancer (OCSC_7, OCSC_8, OCSC_9) in comparison with other groups of cells: small stem cells from “healthy” ovaries (OSC_4, OSC_5, OSC_6), human embryonic stem cells (ESC_10, ESC_11, ESC_12), fibroblasts (FB_13, FB_14. FB_15), and the whole ovarian cell culture from “healthy” ovaries without sorting the small putative stem cells (S_1, S_2, S_3). OCSCs were quite comparable to ESCs for a wide range of these genes, while they were completely different from all other types of cells.
