# Supplementary figures and images for: Novel population of small tumour-initiating stem cells in the ovaries of women with borderline ovarian cancer
Source: Sci Rep. 2016 Oct 5;6:34730. doi: 10.1038/srep34730 (PMC5050448; doi:10.1038/srep34730)

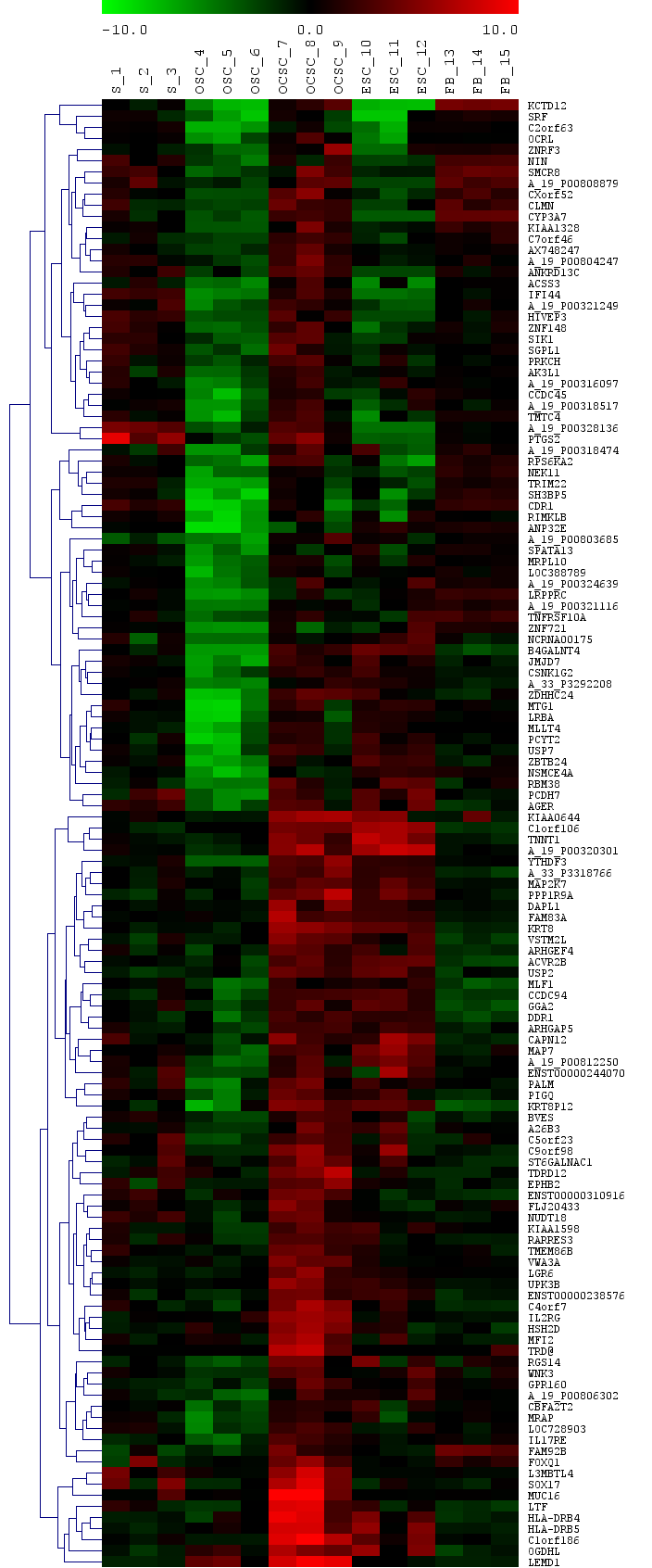

Supplement: Supplementary File 2 [file srep34730-s5.zip › Supplementary file 2-Virant-Klun.png]

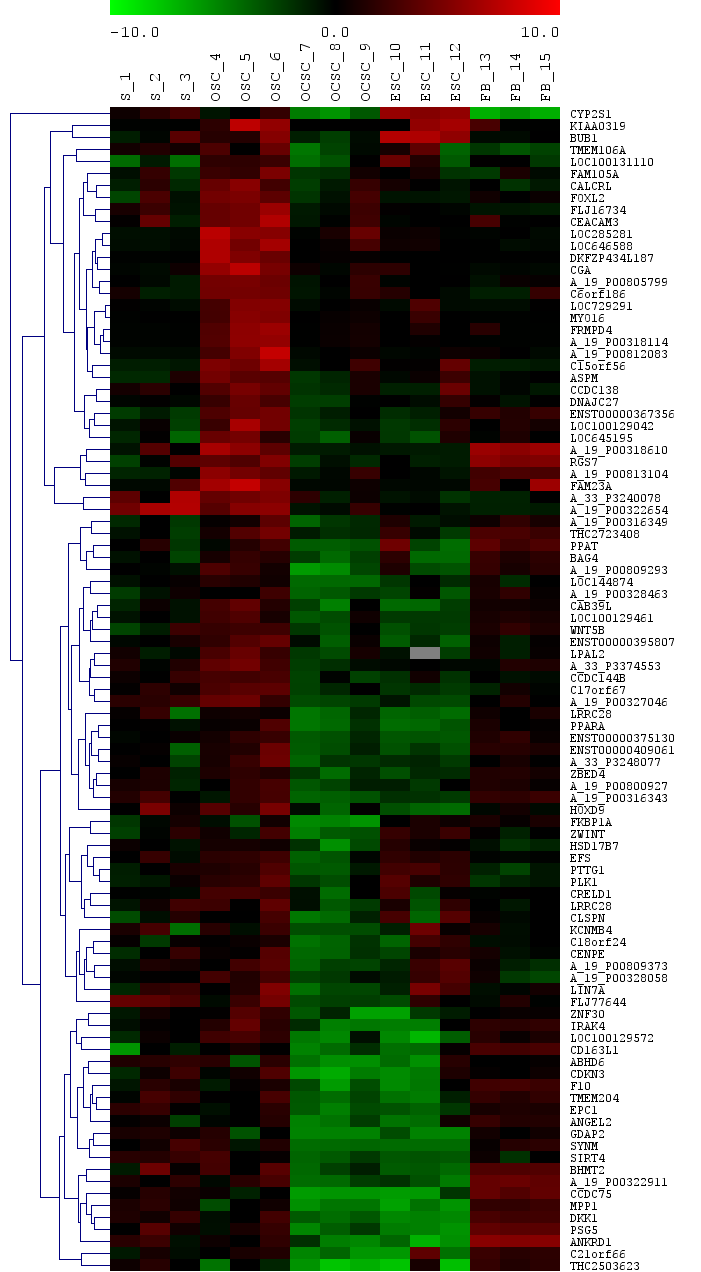

Supplement: Supplementary File 3 [file srep34730-s6.zip › Supplementary file 3-Virant-Klun.png]
